# A Sex-Specific Minimal CpG-Based Model for Biological Aging Using *ELOVL2* Methylation Analysis

**DOI:** 10.3390/ijms26073392

**Published:** 2025-04-04

**Authors:** José Santiago Ibáñez-Cabellos, Juan Sandoval, Federico V. Pallardó, José Luis García-Giménez, Salvador Mena-Molla

**Affiliations:** 1 Department of Physiology, Faculty of Pharmacy, University of Valencia, 46100 Burjassot, Spain; j.santiago.ibanez@uv.es; 2 EpiDisease S.L. (Spin-Off from the CIBER-ISCIII), Parc Científic de la Universitat de Valencia, 46980 Paterna, Spain; 3 Health Research Institute Hospital La Fe (IIS La Fe), 46026 Valencia, Spain; epigenomica@iislafe.es; 4 Department of Physiology, Medicine and Dentistry School, University of Valencia, 46010 Valencia, Spain; federico.v.pallardo@uv.es (F.V.P.); j.luis.garcia@uv.es (J.L.G.-G.); 5 Consortium Center for Biomedical Network Research on Rare Diseases (CIBERER), Institute of Health Carlos III, 46010 Valencia, Spain; 6 INCLIVA Biomedical Research Institute, 46010 Valencia, Spain

**Keywords:** aging, epigenetics, DNA methylation, epigenetic clocks, biological age

## Abstract

Significant deviations between chronological and biological age can signal the early risk of chronic diseases, driving the need for tools that accurately determine biological age. While DNA methylation-based clocks have demonstrated strong predictive power for biological aging determination, their clinical application is limited by several barriers including high costs, the need to analyze hundreds of methylation sites using sophisticated platforms and the lack of standardized measurement tools and protocols. In this study, we developed a multivariate linear model using the analysis of eight CpGs within the promoter region of the very long chain fatty acid elongase 2 gene (*ELOVL2*). The model generated predicts biological age with a mean absolute error (MAE) of 5.04, providing a simplified, cost-effective alternative to more complex methylation-based clocks. Additionally, we identified sex-specific biological clocks, achieving MAEs of 4.37 for males and 5.38 for females, highlighting sex-related molecular differences in the methylation of this gene during aging. Our minimal CpG-based clock offers a practical solution for estimating biological age, with potential applications in clinical practice for assessing age-related disease risks and providing personalized healthcare interventions.

## 1. Introduction

Aging is a natural and gradual process characterized by deterioration of physiological functions and increased vulnerability to disease over time. It encompasses molecular, cellular, and organismal changes leading to decreased tissue function, heightened susceptibility to stress, and a greater risk of age-related diseases. These diseases often include other age-associated processes and conditions such as reduced immune defense, cardiovascular disease, cancer, neurodegenerative disorders, and musculoskeletal diseases (such as arthritis or osteoporosis) [1]. Therefore, aging itself is a significant factor contributing to the onset of most diseases present in older adults. The concept of ‘biological age’ was first introduced in 1947 by H. Benjamin [2] who published one of the first papers indicating that biological age could provide a more accurate measure of an individual’s ageing than chronological age, which only reflects the time elapsed time since birth.

It is now well established that aging is influenced by genetic, environmental, and lifestyle factors, as well as epigenetic mechanisms regulating gene expression and cellular function, which contribute to differences between sexes [3] and impact the acceleration of aging [4,5]. Several tools have been developed to measure biological age using a variety of approaches, such as histology-based data, metabolomics, proteomics, and DNA methylation. Other tools incorporate multiple biomarkers, including clinical variables obtained from blood analysis, hematology, anthropometry, organ function tests, functional aging indices, and frailty indices [6,7]. One of the first molecular strategies developed to assess the biological clock was measuring the length of telomeres: repetitive DNA structures at the ends of chromosomes that shorten with each cell division cycle, a mechanism associated with cellular ageing and health status [8]. Although various methods have been developed to measure telomere length, these exhibit a variability that directly affects the results [9,10]. Moreover, some epidemiological studies have shown contradictory results regarding the relationship between telomere shortening and age [7].

Current approaches based on the exploration of epigenetic clocks have demonstrated a potential to generate more accurate biological age predictors, as most of these clocks correlate well with chronological age [11]. Some DNA methylation clocks (e.g., Horvath, Hannum and Levine) are based on methylation levels at cytosine followed by guanine nucleotide (CpG) sites, while other clocks are based on histone modifications (e.g., the Rechsteiner clock) or miRNA expression levels (e.g., the Huan clock); some are currently being used as biomarkers to predict morbidity and mortality [12,13,14]. Research on DNA methylation has shown that specific regulatory regions of several genes and promoters become progressively methylated with age, indicating a strong functional link between age and DNA methylation [15].

Despite the availability of various tools for measuring biological age, these technologies are not yet widely used in clinical practice due to the need for further clinical applications and validation in clinical trials with larger cohorts, as well as their high cost [16], which, in turn, limits the design of these much-needed bigger clinical trials. To address this barrier, there is currently growing interest in developing clinical epigenetic clocks that use a limited number of CpG sites for age prediction. In this context, methylation levels of CpG islands within the promoter region of the very long chain fatty acid elongase 2 gene *ELOVL2* gene, coding for a transmembrane protein involved in the synthesis of long (C22 and C24) omega-3 and omega-6 polyunsaturated fatty acids (PUFA), have been linked to chronological age across diverse populations, cell types, and tissues [17,18]. Interestingly, ELOVL2 is an enzyme that elongates long-chain omega-3 and omega-6 polyunsaturated fatty acids (LC-PUFAs), precursors of 22:6n-3, docosahexaenoic acid (DHA), and very-long-chain PUFAs [19], which reduces inflammation and has been proposed as a molecule that promotes healthy aging [20]. This is noteworthy as PUFAs play a key role in key biological functions including energy production, modulating inflammation, and maintaining cell membrane integrity.

Further studies by Jung and colleagues have confirmed the correlation between *ELOVL2* methylation and age across various biospecimens, including blood, saliva, and buccal samples. They also investigated methylation levels in other genes such as *KLF14* and *TRIM59*, showing consistent age prediction models across different tissues [21]. Similarly, Slieker et al. analyzed DNA methylation data from multiple tissues and identified tissue-dependent methylation changes, with *ELOVL2* methylation varying between tissues. For example, cerebellar and other brain tissues exhibited low methylation levels compared to the skin, in which methylation increased with age [17].

Methylation evaluation of the *ELOVL2* gene can be used as a quantitative measure of biological aging, serving as a simplified or minimal clock for age prediction. This approach could help reduce costs and facilitate the assessment of biological aging in clinical trials using large patient cohorts. Predicting biological age in this way could indicate whether individuals are experiencing healthy aging or are in an accelerated aging state compared to their chronological age. One of the key challenges with these clocks is ensuring robustness. In this study, we propose a potentially simplified and robust biological clock, which could provide a more practical and reliable measurement of biological age for widespread use.

## 2. Results

### 2.1. Reproducibility and Robustness

Reproducibility analysis, consisting of evaluating three samples from the same person collected simultaneously, showed that methylation percentages for the nine CpG sites of the three samples had excellent reproducibility, as evidenced by the low the coefficient of variation (CV) [Table 1]. Mean methylation values for CpG sites ranged from 44.09 to 88.28%, with CpG3 and CpG7 showing the highest mean values (83.03% and 88.28%, respectively). The SD for these sites were low, ranging from 0.60% to 3.01%, indicating minimal variation between samples. Notably, the CVs for the CpG sites were remarkably low, with the highest at 5.27% for CpG1 and the lowest at 0.85% for CpG4, signifying great precision in the measurements. This consistency across samples underscores the robust reproducibility of the assay [22], confirming its reliability for detecting DNA methylation with minimal experimental variability. These results strongly indicate that the method used provides highly reproducible methylation measurements, a critical factor for reliable downstream analysis and interpretation.

Table 2 shows the results obtained in the stability approach after 12 months of storage. The methylation levels in the nine CpG sites showed good reproducibility, demonstrating low variability in general. Specifically, five CpG sites (CpG1, CpG3, CpG4, CpG6, and CpG7) exhibit low CVs, below 10%, indicating high reproducibility. CpG2, CpG8, and CpG9 showed CV values between 10% and 20%, which are still considered acceptable and indicate good representability of the data [23]. CpG5 showed a CV of 20.39%, which was slightly higher than the ideal threshold but remained within an acceptable range. Overall, the low standard deviations and CVs across most sites highlighted the robustness of the pyrosequencing method used, demonstrating consistent and reliable measurements of DNA methylation levels. The validity of these data is supported by the high level of reproducibility obtained.

### 2.2. Methylated CpGs Correlation

The individual linear regression models showed a moderate-to-strong positive correlation between methylation levels and chronological age observed across CpGs (Table 3), with R coefficients ranging from 0.6538 to 0.8534. The R^2^ ranged between 0.4274 and 0.7455, indicating that approximately 42.74–74.55% of the variance in methylation levels could be explained by chronological age (Supplementary Figure S1). The adjusted R^2^ ranged from 0.4203 to 0.7423, adjusting for the number of predictor variables in the model. The standard errors ranged from 2.9012 to 8.0435, reflecting the average deviation of observed methylation values from the predicted values. Mean absolute error (MAE) values exhibit a range from 7.54 in the linear model CpG7 to a higher value of 13.57 in the model with CpG1. This indicates that the model with CpG7 has the best predictive accuracy in terms of MAE. The root mean square error (RMSE) values varied between 9.63 and 19.07, reflecting the overall dispersion of prediction errors across the different models. *p*-values were all highly significant, indicating strong statistical support for the correlation between chronological age and methylation level for each chronological age range of the training group. The best individual linear regression model was CpG7 with R^2^ 0.7423 (Table 3).

**Figure 1:**
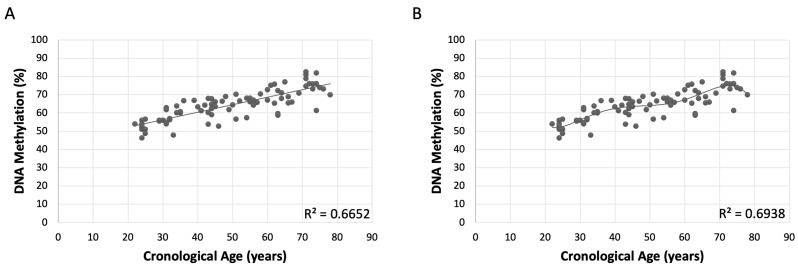
Graphical representation of the non-sex-specific models. (**A**) Linear model of the mean of all CpG values. (**B**) Polynomial model of the mean of all CpG methylation values. The X-axis represents the chronological values in years, while the Y-axis represents the percentage of DNA methylation. The metric includes the coefficient of determination (R^2^).

Likewise, the linear regression model using the mean of all CpG methylation values showed a strong positive correlation (R = 0.8156) between methylation levels and chronological age, with R^2^ of 0.6652 and an adjusted R^2^ of 0.6610, explaining approximately 66.52% of the variance (Figure 1A). The standard error was 4.8526, MAE was 9.42, RMSE was 11.94, and the *p*-value was highly significant (6.13 × 10^−21^) [Table 3].

The polynomial regression model showed a strong positive correlation (R = 0.6938) and explained approximately 69.38% of the variance in methylation levels (R^2^ = 0.6938) (Figure 1B). Adjusted R^2^ was 0.6739, and the residual standard error was 4.759. The model’s MAE and RMSE were 7.31 and 9.35, respectively. The *p*-value was 2.20 × 10^−16^, indicating strong statistical significance (Table 3). Diagnostic tests show no systematic patterns in the residuals, confirming an adequate model fit. The Breusch–Pagan test was non-significant (*p* = 0.4982), supporting homoscedasticity, and the Q-Q plot (Supplementary Figure S2) displayed minor deviations from normality, within acceptable limits for model reliability.

After evaluating various regression models to understand the relationship between methylation levels and age across CpGs, the CpG7 model showed the highest individual correlation, but the model with all CpGs provided a better overall understanding. This is because methylation values were not as high as in the CpG7 model (Supplementary Figure S1 and Figure 1A). Therefore, considering its higher explanatory and strong statistical significance, the polynomial regression model of degree 5 emerged as the optimal choice for modeling the relationship between methylation levels and age across all CpGs (Figure 1B and Table 3).

We performed multivariate linear models for all possible combinations from 1 to nine CpGs (Supplementary Table S1). The best results were achieved by performing multivariate linear models using different combinations of CpGs (Table 4). Among all the models evaluated, the best was the model constructed using eight CpGs (CpGs 1, 2, 3, 4, 6, 7, 8, and 9) which stood out as the most robust (Supplementary Table S2). This model did not include CpG5, which showed the highest CV variation. The model displayed a very strong overall fit (R^2^ = 0.867) with an adjusted R^2^ of 0.852, indicating that these CpGs collectively explain 86.70% of the variance in the outcome variable. The standard error was 6.365, the MAE was 4.55, and the RMSE was 6.01, with a highly significant *p*-value of 2.48 × 10^−29^ (Table 4).

The next most relevant model was the one using nine CpGs (CpGs 1, 2, 3, 4, 5, 6, 7, 8, and 9). This model also showed a high fit (R^2^ = 0.867) and an adjusted R^2^ of 0.850, like the eight CpGs model, with a standard error of 6.405, MAE of 4.55, and RMSE of 6.00. The *p*-value was significant at 1.93 × 10^−28^ (Table 4).

**Figure 2:**
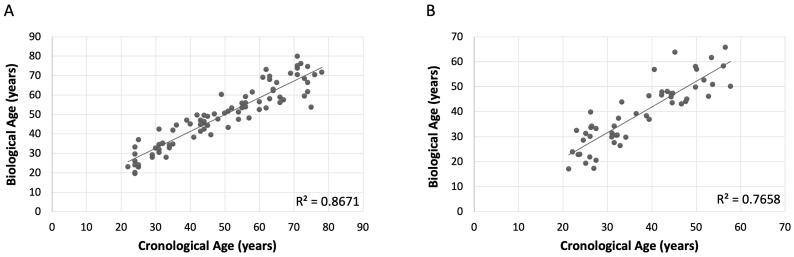
Graphical representation of the non-sex-specific training cohort obtained with a linear multivariate model with eight CpGs (CpG1, CpG2, CpG3, CpG4, CpG6, CpG7, CpG8, CpG9). (**A**) Linear multivariate model obtained in the discovery cohort. (**B**) Linear multivariate model obtained in the validation cohort. The X-axis represents chronological values in years, while the Y-axis represents predicted values in years. The metric includes the coefficient of determination (R^2^).

Considering all models generated, the multivariate linear model using the selected eight CpGs provided the strongest fitness and captured the most variance in the outcome variable, closely followed by the nine CpGs model (Table 4). Models with fewer CpGs showed a progressive decrease in the adjusted R^2^, indicating a reduction in explanatory power as predictors are removed. To maximize predictive accuracy and understand the collective influence of DNA methylation, the model including eight CpGs was the preferred choice (Figure 2A).

Using the equation derived from the multivariate linear model, the results obtained with the validation cohort demonstrated a good performance for the assay. The multiple correlation coefficient (R) of 0.878 and the coefficient of determination (R^2^) of 0.7709 indicated a robust relationship between the methylation levels and age, explaining approximately 77.09% of the variance. The adjusted R^2^ of 0.766 further supported this relationship. The standard error of 6.1994 reflected that the model’s predictions obtained a comparable accuracy to the training model. Additionally, the ANOVA confirmed the statistical significance of the validation model, with a *p*-value significantly below 0.001 (Figure 2B and Table 5).

Despite slight differences in performance metrics between the training and validation models, the consistency in results suggested that the training model is validated by the validation results (Table 5). The high correlation coefficients, significant R^2^ values, and low standard errors in both models, indicate that they effectively captured the relationship between predictor variables and outcome. Therefore, the training model appeared to fit well to the new data, providing confidence in its predictive capability.

We further explored the models by separating female and male samples, calculating multivariate linear models for each sex. All possible sex-specific models were run, from all nine CpGs together to a single CpG for each one. The rationale of this assay was to find out whether any CpGs were associated with either sex, and whether age prediction could be improved specifically for each sex.

**Figure 3:**
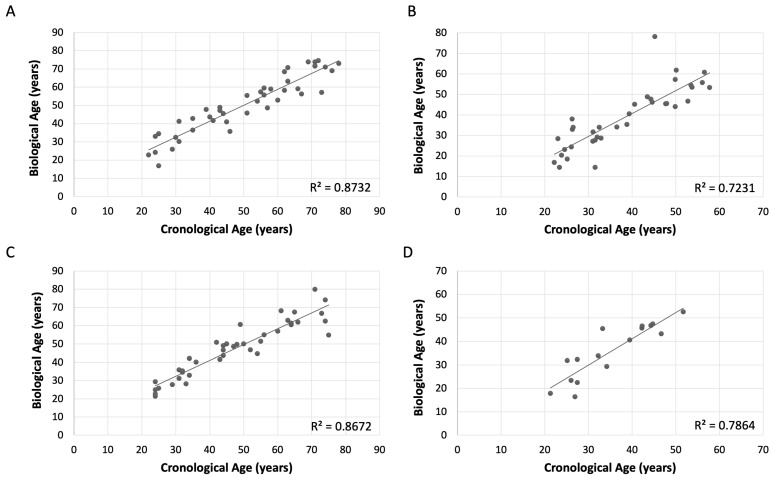
Graphical representation of the sex-specific linear multivariate model. (**A**) Graphical representation of the female training cohort obtained with a linear multivariate model with six CpGs (CpG1, CpG4, CpG6, CpG7, CpG8, CpG9). (**B**) Graphical representation of the female validation cohort obtained with a linear multivariate model with six CpGs. (**C**) Graphical representation of the male training cohort obtained with a linear multivariate model with six CpGs (CpG1, CpG2, CpG3, CpG7, CpG8, CpG9). (**D**) Graphical representation of the male validation cohort obtained with a linear multivariate model with six CpGs. The X-axis represents chronological values in years, while the Y-axis represents predicted values in years. The metric includes the coefficient of determination (R^2^).

The results revealed significant findings, particularly highlighting the models using six CpGs, which exhibit the best overall fit for females (Table 6), specifically the model with six CpGs (CpG1, CpG4, CpG6, CpG7, CpG8, CpG9) [Supplementary Table S3]. This model demonstrated a multiple correlation coefficient of 0.9344 and an adjusted R^2^ of 0.8521, indicating a strong explanatory power (Figure 3A). The standard error for this model is 6.52, with a MAE of 4.75 and a RMSE of 5.96. Similarly, for males (Table 7), the six CpGs model (CpG1, CpG2, CpG3, CpG7, CpG8, CpG9) [Supplementary Table S4] showed a multiple correlation coefficient of 0.9312 and an adjusted R^2^ of 0.8430, reflecting its robustness (Figure 3C). The standard error for the male model was 6.41, with a MAE of 4.22 and a RMSE of 5.82. These metrics underscore the precision and reliability of the six CpGs models for both sexes. Interestingly, the different CpGs involved in each model meant that sex-associated differences could be identified from the results. It was observed that CpG4 and CpG6 were specific for females (Supplementary Table S3), while CpG2 and CpG3 were specific for males (Supplementary Table S4).

Overall, the analysis showed a gradual decrease in the adjusted R^2^ as the number of CpGs reduced from 9 to 1. For females, the nine CpGs model started with an adjusted R^2^ of 0.845, which decreased to 0.776 in the one CpG model (Table 6). For males, the nine CpGs model began with an adjusted R^2^ of 0.839, dropping to 0.711 in the one CpG model (Table 7). This trend highlights the importance of including multiple CpGs to enhance model accuracy and reliability. The six CpGs models stand out for their superior adjusted R^2^ values, confirming their effectiveness in capturing the underlying relationships in the data for both females and males.

The multivariate linear models were validated in an independent cohort for both females and males (Table 8), demonstrating a good performance for multivariate models. For the female validation cohort, the R was 0.8503, with an R^2^ of 0.7231, explaining 72.31% of the variance (Figure 3B). The adjusted R^2^ was 0.7149, which although slightly lower than that of the training cohort, still indicates a strong relationship. The standard error was 6.1329, with a MAE of 5.38 and a RMSE of 7.93. The *p*-value for the validation cohort was significantly below 0.001, supporting the model’s validity. In the male validation cohort (Table 8), the R was 0.8867, with a R^2^ of 0.7863 (Figure 3D). The adjusted R^2^ was 0.7711, with a standard error of 4.413, a MAE of 4.37, and a RMSE of 5.33. The *p*-value was significantly below 0.001, further supporting the model’s validity.

Despite slight differences in the performance metrics between the training and validation models, the results suggested that the training models were confirmed by the validation model results (Table 8). The high correlation coefficients, significant R^2^ values, and low standard errors in both models indicated that they effectively captured the relationship between the predictor variables and outcome (Figure 3). Consequently, the training models demonstrated robust generalization to new data, instilling confidence in their predictive accuracy.

Finally, the samples were classified into age categories (20–29, 30–39, 40–49, 50–59, 60–69, 70–79 years), and the performance of the best models (eight CpGs non-sex-specific for all samples, six CpGs for females, and six CpGs for males) was analyzed.

For the eight CpG non-sex-specific model using all samples, the MAE ranged from 3.09 to 7.01, and the RMSE ranged from 4.16 to 9.36 across different age groups (Table 9). The overall MAE and RMSE for this model were 4.55 and 6.01, respectively. These values indicated that the model performed reasonably well, but the errors increased significantly in older age groups, particularly in the samples obtained from subjects with ages in the 70–79 range.

In contrast, the models tailored for each sex demonstrated a more consistent performance across age groups. For the 6 CpG female model, the MAE ranged from 3.37 to 5.97, and the RMSE ranged from 4.16 to 6.65 (Table 9). The overall MAE and RMSE for this model were 4.75 and 5.96, respectively. This model showed a slightly better performance for middle-aged females compared to younger and older age groups.

Similarly, the six CpG male model exhibited a MAE range of 2.04 to 9.40 and a RMSE range of 2.57 to 11.50 (Table 9). The overall MAE and RMSE for the male model were 4.22 and 5.82, respectively. Notably, this model performed exceptionally well in younger age groups (20–29) but showed a significant increase in the error for the oldest age group (70–79).

The sex-differential six CpGs models exhibited more consistent error metrics across different age groups compared to the eight CpG non-sex-specific model, which included all samples (Table 9). The tailored models display similar MAE and RMSE values, suggesting that sex-specific models may offer more precise age predictions by reducing the variability seen in the model using combined samples. The errors were notably higher in the oldest age group across all models, indicating a potential area for further refinement.

## 3. Discussion

Epigenetics has emerged as a key mechanism underlying the molecular processes that contribute to aging [24]. For this reason, characterization of epigenetic clocks is gaining importance because of its potential to evaluate biological aging, establish health status [13,14], and assess the effects of interventions aimed at promoting healthy aging [14,25]. In this context, we have recently described an epigenetic clock based on miRNAs to evaluate the biological age of skin [26].

Among the different mechanisms of epigenetic regulation, DNA methylation represents a remarkably stable epigenetic signature. During aging, a global age-associated hypomethylation has been observed, however, the process has also been reported in specific loci, including tumor suppressor genes and Polycomb target genes [1,27].

The promoter of the *ELOVL2* gene is one of the most informative genes related to human epigenetic age [28]. *ELOVL2* methylation has been shown to strongly correlate with biological age in humans [29], as well as in rodents [20,30]. Furthermore, silencing *ELOVL2* through methylation or ELOVL2 protein depletion accelerates aging in the mouse retina [31,32] and has been linked to diabetes [33], as well as increased risk of breast and male colorectal cancer [34]. Among other potential applications, this epigenetic marker can be used for predicting the development of neurodegenerative diseases, as alterations in the *ELOVL2* methylation status have been observed in patients at early stages of Alzheimer’s disease [35]. Therefore, methylation of the *ELOVL2* gene promoter can be considered a good indicator of aging, frailty and age-associated conditions.

In this study, we used DNA from buccal swabs of healthy people for age prediction using the simplified ELOVL2 epigenetic clock. Buccal swabs were chosen because they offer a simple, painless, and non-invasive method for DNA collection, and are easier to transport and store. Collection can be performed by the donor themselves or by a non-specialist, whereas obtaining blood samples is a more invasive procedure requiring a trained professional to draw the blood. Moreover, use of this DNA source is supported by various studies that report a chronological age-related increase in *ELOVL2* gene methylation across different tissues, including blood, saliva, and buccal swabs [36,37,38]. Indeed, age prediction accuracy (R^2^ and MAE) for buccal swabs resulted comparable to blood and saliva. Therefore, DNA from buccal swab is an optimal sample type for studying biological aging using *ELOVL2* methylation as an epigenetic clock.

At the technical level, we found significant variability in the experimental design of previous studies evaluating epigenetic clocks, particularly concerning sample size and DNA sources used (i.e., skin, blood, buccal epithelium, etc.). Some studies used a relatively small number of DNA samples, such as Richards et al., who used a total of 28 peripheral blood samples [38] and the study by Kampmann et al., which used 49 DNA samples obtained from blood [39]. In contrast, other works used larger sample sizes, such as Horvath et al., who included 485 DNA samples from skin and blood [40] and the study by McEwen et al., which included 1721 DNA samples from buccal epithelium [41]. According to Mayne et al., an epigenetic clock should ideally be calibrated with a minimum of 70 samples, but a sample size of 134 individuals would yield more precise and accurate models for predicting epigenetic age [42].

Several studies, such as those by El-Shishtawy et al. and Sukawutthiya et al., have used 100 blood samples to construct epigenetic clocks [43,44]. Our epigenetic clock, based on *ELOVL2* methylation analysis using the pyrosequencing approach, was developed with 83 samples for training, exceeding the minimum threshold proposed by Mayne et al. [42]. Validation was conducted with a relatively small sample size (*n* = 52), following the guidelines proposed by Archer et al. [45]. This brings the total sample size to 135, which we consider sufficient for generating a robust epigenetic clock. The strong predictive performance of the model, reflected in its high adjusted R^2^ and low error metrics, supports its validity as a reliable approach for age estimation.

Regarding the number of CpG used, current research has generated epigenetic clocks using large quantities [46,47]. However, other studies have used fewer CpGs, sometimes combining them with CpGs from other genes [38]. There are even studies describing epigenetic clocks using only two CpG sites [44] or just one [48]. It is therefore feasible to design epigenetic clocks using the smallest possible number of CpGs, focusing on the ones showing the most relevant changes over chronological age to provide maximum information while also reducing unnecessary costs. Indeed, some studies have reported ELOVL2-based clocks using a minimal number of CpG sites that still provide significant information [43,44]. In our case, we aimed to identify the most robust CpGs that provided more information for our models, resulting in a total of eight CpGs sites for the non-sex-specific model, and six CpGs for both female and male models.

An important aspect of our study was the technical validation carried out. We analyzed variability within the same individual and the changes in DNA methylation in a stored sample over 12 months. Few studies have conducted these types of technical validations, which are necessary to assess the robustness and performance of any analytical determination. Kampmann et al. evaluated inter-laboratory reproducibility by performing validations across different laboratories [39], while Zbieć-Piekarska et al. tested reproducibility between two laboratories and the stability of epigenetic analysis over time [37]. Their study conducted temporal comparisons of bloodstains stored on tissue paper after 5, 10, and 15 years at room temperature conditions, with age prediction success rates ranging from 60–78%. Our results align with these previous studies, showing minimal CV, indicating good reproducibility for all CpG sites. Among the different CpGs analyzed, CpG5 exhibited the highest CV value and was subsequently eliminated from the models. Based on the CpG sites selected for the models, we can confirm good reproducibility and sample stability, even after one year of storage.

We obtained three models for epigenetic age prediction. The first was the non-sex-specific model using eight CpGs (CpG1, CpG2, CpG3, CpG4, CpG6, CpG7, CpG8, CpG9) showing an R^2^ adj 0.852, MAE 4.55 and RMSE 6.01. These results suggest the model performs well for age prediction.

Interestingly, unlike other studies which observed no significant differences between males and females [43,49,50], we found that some CpGs performed better in the sex-specific models generated for age prediction. As a next step, we stratified the training model samples by sex and then adjusted the models for each one. After analyzing the results, we observed that the best models for each sex used six CpGs, but the two models did not share the same CpGs. In the case of females, the best predictors of chronological age of control subjects were CpG1, CpG4, CpG6, CpG7, CpG8, CpG9, whereas for males the best CpGs fitted in the model were CpG1, CpG2, CpG3, CpG7, CpG8, CpG9. Therefore, two different CpGs (CpG4 and CpG6 for females and CpG2 and CpG3 for males) were used to construct the sex-related models for subsequent age prediction.

Recent advances in epigenetic aging research have highlighted the importance of accounting for sex-specific differences in methylation patterns. Although *ELOVL2* methylation has long been recognized as a robust marker of chronological aging [28,31], emerging studies indicate that the rate and pattern of epigenetic changes differ between sexes. Kankaanpää et al. provided compelling evidence that epigenetic clocks yield distinct biological age estimates for men and women, potentially reflecting variations in hormonal milieu, lifestyle, and genetic factors [5]. Likewise, Yusipov et al. reported that males exhibit a markedly higher age-associated increase in methylation variability than females [51]. Our sex-specific minimal CpG-based model, which leverages *ELOVL2* methylation, extends these observations by capturing subtle yet biologically relevant differences in the epigenetic aging trajectories of men and women. This refined approach not only enhances the accuracy of age estimation but also underscores the potential for personalized aging interventions aimed at mitigating sex-specific health disparities.

The adjusted R^2^ value for the six CpG female model was 0.852 and for the six CpGs male model 0.843, which were similar to that obtained for the overall eight CpGs non-sex-specific model (R^2^ adj 0.852, MAE 4.55, RMSE 6.01). However, in the case of the six CpGs male model the MAE (4.22) and RMSE (5.82) improved compared to the eight CpGs non-sex-specific model, and the in six CpGs female one the value of RMSE (5.96) was reduced and the values of MAE (4.75) were homogeneous: that is, they were more similar in the different subgroups. Thus, the models obtained from differentiating between sexes offer better results to predict biological age than those not segregated by sex.

When we evaluated the age prediction values along the different age ranges for the eight CpGs non-sex-specific model, the subgroup of 20 to 29 years old showed a MAE value of 3.67, while in the 70 to 79 years old subgroup a MAE value of 7.01 was observed. In contrast, in the six CpGs female model, the subgroup of females aged 20 to 29 years old exhibited a MAE value of 5.09, whereas in females aged 70–79 years old we observed a MAE value of 7.01. Particularly in the case of the six CpGs male model, the samples obtained from males aged 70–79 years old produced a higher MAE value (9.40) and RMSE (11.49).

All models showed excellent adjusted R^2^ values, which concur with or even surpass those reported in different published studies in which sex was not taken into account in generating specific models for age prediction [37,44,52]. MAE and RMSE values also indicated good performance. Hanafi et al. published a systematic analysis including different models and observed different MAE values, ranging from 0.33 to 7.01 [37,44,52,53]. Our results fall within those ranges, particularly our models ranged on intermediate values described by Hanafi et al. [53].

When evaluating our results, we observed that the deviation in the MAE was greater in older subjects. This suggests that our models perform less accurately as chronological age increases. One possible explanation for the higher MAE observed in older cohorts is that, with aging, comorbidities tend to arise, or individuals may be affected by undiagnosed age-related conditions that have not yet manifested [53]. Recent studies have highlighted the association between *ELOVL2* methylation and age-related pathologies, further supporting this hypothesis. For instance, ELOVL2 deficiency has been linked to age-related macular degeneration phenotypes in human retinal pigment epithelium cells [53], suggesting that methylation patterns may be influenced by underlying age-related conditions, potentially affecting prediction accuracy in older individuals. Similarly, research on chronic kidney disease has explored the role of *ELOVL2* methylation in renal and cardiovascular events [54], indicating a possible interplay between this epigenetic marker and age-related health deterioration. Although independent associations were not found after adjusting for covariates, this study underscores the complex relationship between *ELOVL2* methylation and comorbidities associated with aging. Additionally, evidence suggests that *ELOVL2* methylation is associated with metabolic dysfunction and mitochondrial stress [20], both of which increase with age and could contribute to greater variability in methylation patterns among older individuals. Taken together, these findings support the theory that undiagnosed age-related conditions may partially account for the observed discrepancies in age prediction accuracy in older cohorts.

The different age prediction models we have generated in this study have also been tested by a validation cohort, demonstrating reliable results for the three models generated, unlike many previous publications [37,39,43] with proposed but unvalidated models. Our validation results showed an adjusted R^2^ of 0.833, with a MAE of 5.04 and an RMSE of 6.26 for the eight CpGs model. In the six CpGs male model we obtained an R^2^ of 0.771 with a MAE of 4.37 and a RMSE of 5.33, while in the six CpGs female model we obtained an R^2^ of 0.714, a MAE of 5.38, and a RMSE of 7.93.

An important opportunity for future improvement in this study is the inclusion of greater diversity within the cohorts used. While the model has demonstrated strong predictive accuracy in estimating biological age, the lack of explicit stratification by ethnic background in the current dataset may limit the generalizability of the results to broader populations. DNA methylation profiles have been shown to vary across ethnic groups due to genetic, environmental, and socioeconomic factors. Therefore, future research would benefit from the inclusion of more diverse cohorts, allowing for a more comprehensive assessment of the model’s accuracy across varying genetic and environmental contexts. Validation in multi-ethnic datasets would not only enhance the robustness of the model but also expand its clinical applicability, ensuring that it performs effectively across a wider range of populations. This perspective provides a valuable direction for further research and clinical applications, contributing to the ongoing refinement and broader implementation of the model.

In conclusion, our analysis of up to nine methylation sites in the *ELOVL2* gene has proven reliable even after one year of storage and has been validated through standard statistical procedures. This allowed us to develop robust models for biological age prediction, including an eight CpGs non-sex-specific model for both sexes and two six CpGs sex-specific models, all showing strong R^2^, MAE, and RMSE values. Based on our findings, we recommend applying sex-specific DNA methylation models for age prediction, as different CpG sites are necessary to optimize accuracy for each sex. Given the performance and cost-effectiveness of this approach, we propose its evaluation in clinical settings to enhance the precision of age prediction in personalized medicine.

## 4. Materials and Methods

### 4.1. Human Samples

A total of 83 DNA samples from buccal swabs of healthy individuals were obtained from the Biobank for Biomedical Research and Public Health of the Valencian Community (IBSP-CV) and used as a training group (Group 1, *n* = 83, 49.2 ± 16.6 years) [Table 10]. All individuals were of self-reported European (Caucasian) ancestry, specifically from the Spanish Mediterranean region. Samples were classified by age range into subjects aged 20–29 years (*n* = 12), 30–39 years (*n* = 14), 40–49 years (*n* = 16), 50–59 years (*n* = 13), 60–69 years (*n* = 15), and 70–79 years (*n* = 13). Another 52 DNA samples from buccal swabs obtained from the Biobank IBSP-CV were used as a validation group (Group 2, *n* = 52, 37.4 ± 10.8 years) [Table 11].

All participants signed the IBSP-CV written informed consent to participate in biomedical research. The Foundation for the Promotion of Health and Biomedical Research of the Valencian Community (FISABIO) ethics committee approved the study (reference number 2022/343). The study was conducted in accordance with the local legislation and institutional requirements.

### 4.2. DNA Purification and Bisulfite Conversion

DNA extraction was performed using the Chemagic™ DNA Buccal Swab Kit H96 (Ref. CMG-748, Perkin Elmer, Waltham, MA, USA) in a Chemagic 360 Instrument (Perkin Elmer, Waltham, MA, USA) according to the manufacturer’s protocol. The extracted DNA was quantified by NanoDrop One Spectrophotometer (ThermoFisher Scientific, Waltham, MA, USA).

DNA concentrations were measured using the Qubit dsDNA HS Assay Kit (ThermoFisher Scientific, Waltham, MA, USA). A total of 500 ng of genomic DNA underwent conversion treatment with bisulfite using the EZ-96 DNA Methylation Kit (D5004 Zymo Research Corp., Irvine, CA, USA) following the manufacturer’s instructions [39].

#### 4.2.1. *ELOVL2* Methylation Analysis Using Bisulfite Pyrosequencing

Specific sets of primers for PCR amplification and sequencing were designed to hybridize with CpG-free sites to ensure methylation-independent amplification, using PyroMark assay design version 2.0.01.15 software (Qiagen, Hilden, Germany) in a region of the *ELOVL2* gene promoter, which includes the nine CpG sites of interest.

PCR was performed under standard conditions with biotinylated primers and the PyroMark Vacuum Prep Tool (Biotage, Uppsala, Sweden) was used to prepare single-stranded PCR products according to the manufacturer’s instructions. PCR products were observed at 2% agarose gels before pyrosequencing.

Reactions were performed in the PyroMark Q24 System version 2.0.6 (Qiagen, Hilden, Germany) using appropriate reagents and protocols, and the methylation value was obtained from the average of the CpG dinucleotides included in the sequence analyzed. Controls to assess correct bisulfite conversion of the DNA were included in each run, as well as sequencing controls to ensure the fidelity of the measurements. The graphic representation of methylation values shows bars identifying CpG sites that present percentage methylation values.

#### 4.2.2. Robustness of the Values

In the following section, we describe the different approaches employed in the study to assess the robustness and consistency of the various methods.

The first step was to check reproducibility. For this purpose, three swabs from the same person at the same time were collected for comparison. DNA extraction, bisulfite treatment, and pyrosequencing of three samples were carried out.

The second step was to check stability. This procedure consisted of extracting three samples that had been frozen for one year. Two swabs were collected from the same individual, one swab was analyzed on the same day, while another swab was stored at −20 °C for one year. After one year, DNA extraction, bisulfite treatment and pyrosequencing of the three samples were carried out as described in the previous sections.

We obtained the mean and standard deviation for the methylation of each CpG to estimate the coefficient of variation (CV), defined as the ratio of the standard deviation to the mean.

#### 4.2.3. Robustness of Values

##### Lineal Regression Models

Age (X-axis) was compared against methylation levels (Y-axis) on average and for each individual CpG in a linear model, performing a total of nine models for each CpG and one model for the mean of all CpGs together [55].

##### Polynomial Regression Models

A polynomial model is a mathematical tool used to describe relationships between variables that may not be linear. Instead of assuming a direct relationship between the variables, a polynomial model allows users to capture more complex patterns such as curves or nonlinear trends. To build a polynomial model, we used data representing the relationship between the independent variable (subject age) and the dependent variable (different methylation levels). Next, we selected the degree of the polynomial that best fit the data: in this case, a polynomial of degree five was used.

To ensure the validity and robustness of the model, several diagnostic analyses were performed. The coefficients of the polynomial were adjusted using the method of least squares, and the model’s performance was validated to ensure it is suitable for the data [55]. Residual versus predicted value plots were examined to evaluate the adequacy of the model’s fit. Q-Q plots were generated to assess the normality of residuals, ensuring that deviations primarily occurred at the extremes while most residuals followed an approximately normal distribution. Additionally, the Breusch–Pagan test was applied to check for homoscedasticity, verifying that the variance of residuals remained stable across different values of the independent variable. These validation steps were conducted to confirm that the model provided a reliable and statistically sound representation of the relationship between DNA methylation levels and age.

##### Linear Multivariate Model

Linear multivariate models are an extension of the linear model, which can be used to make a prediction for a given observation based on its pattern of covariates, the value of a continuous variable, or the probability of occurrence of a dichotomous variable. In our case, subjects’ biological age was used as the independent variable, and the nine different CpGs were used as dependent variables. Multiple multivariate linear models were generated, the first including all the predictor characteristics (CpGs). Subsequent models were built by removing the CpGs with least statistical value identified in the previous models for each new one, until arriving at a final model with only one CpG. The linear regression coefficient between age and methylation level was presented with the R^2^ [55].

### 4.3. Statistical Analysis

A statistical analysis and graphical representations were performed using MS Excel (Microsoft) to compute the mean absolute error (MAE) and root mean square error (RMSE). All continuous data were analyzed for normal distribution. Data were presented as mean and standard error (SEM) for quantitative parametric data, with a 95% confidence interval (CI).

The MAE is a measure of the accuracy of a prediction model. It is used to quantify the average magnitude of errors in the predictions made by the model, without considering their direction (positive or negative). The RMSE is a measure used to quantify the discrepancy between the values predicted by a model and the actual observed values in a dataset. It is calculated as the square root of the difference between the predicted value and the actual value. Data variation was evaluated by considering the MAE and RMSE using a difference between an age value of observed DNA samples and predicted values. The correlation analyses were assessed using the coefficient of multiple correlation (R), coefficient of determination (R^2^), and adjusted coefficient of determination (Adjusted R^2^). R represents the strength and direction of the relationship between predictor variables and the response variable, while R^2^ indicates the proportion of the variance in the response variable explained by the predictor variables. Adjusted R^2^ provides a more accurate estimate of the proportion of variance explained, considering the number of predictor variables in the model. The standard error reflects the accuracy of the regression coefficients, while the *p*-value assesses the significance of the observed relationships between predictor variables and the response variable. *p*-values: **** *p*-value  <  0.0001, *** *p*-value  <  0.001, ** *p*-value  <  0.01, or * *p*-value  <  0.05 were considered statistically significant.

(1) $$MAE=\frac{å|Predicted\;age-Chronological\;age|}{n}$$

(2) $$RMSE=\sqrt{\frac{{å\left(Predicted\;age-Chronological\;age\right)}^{2}}{n}}$$

## Figures and Tables

**Table 1:** Methylation levels obtained from pyrosequencing three samples from the same person. The methylation values for each CpG in percentage (%), the mean, the standard deviation (SD), and the coefficient of variation (CV) are indicated.

Sample ID	CpG1 (%)	CpG2 (%)	CpG3 (%)	CpG4 (%)	CpG5 (%)	CpG6 (%)	CpG7 (%)	CpG8 (%)	CpG9 (%)
Sample 1	53.64	53.55	81.16	69.66	50.99	79.12	87.33	44.2	58.39
Sample 2	58.71	58.23	84.38	70.77	53.08	81.33	89.72	43.48	61.24
Sample 3	58.98	57.09	83.54	70.59	49.42	78.08	87.8	44.6	59.75
Mean	57.11	56.29	83.03	70.34	51.16	79.51	88.28	44.09	59.79
SD	3.01	2.44	1.67	0.60	1.84	1.66	1.27	0.57	1.43
CV	5.27	4.34	2.01	0.85	3.59	2.09	1.43	1.29	2.38

**Table 2:** Methylation levels obtained from three different samples after 12 months in the stability approach, indicating the methylation values for each CpG, the mean, the standard deviation (SD), and the coefficient of variation (CV).

Sample ID	CpG1 (%)	CpG2 (%)	CpG3 (%)	CpG4 (%)	CpG5 (%)	CpG6 (%)	CpG7 (%)	CpG8 (%)	CpG9 (%)
0 Months Sample 1	53.85	53.06	84.02	68.3	49.5	77.94	87.25	45.2	60.15
12 Months Sample 1	48.31	45.41	80.49	62.42	43.16	75.14	85.82	41.19	58.12
Mean	51.08	49.24	82.26	65.36	46.33	76.54	86.54	43.20	59.14
SD	3.92	5.41	2.50	4.16	4.48	1.98	1.01	2.84	1.44
CV	7.67	10.99	3.03	6.36	9.68	2.59	1.17	6.56	2.43
0 Months Sample 2	34.54	52.72	75.79	66.72	48.00	82.81	87.7	37.18	50.28
12 Months Sample 2	58.05	39.8	93.67	91.35	95.91	98.53	97.37	42.36	93.4
Mean	46.30	46.26	84.73	79.04	71.96	90.67	92.54	39.77	71.84
SD	16.62	9.14	12.64	17.42	33.88	11.12	6.84	3.66	30.49
CV	35.91	19.75	14.92	22.04	47.08	12.26	7.39	9.21	42.44
0 Months Sample 3	49.15	49.81	81.58	63.13	43.08	75.07	85.14	39.2	53.34
12 Months Sample 3	51.22	48.7	80.22	63.16	40.47	72.95	82.78	37.35	55.49
Mean	50.19	49.26	80.90	63.15	41.78	74.01	83.96	38.28	54.42
SD	1.46	0.78	0.96	0.02	1.85	1.50	1.67	1.31	1.52
CV	2.92	1.59	1.19	0.03	4.42	2.03	1.99	3.42	2.79
Mean	49.19	48.25	82.63	69.18	53.35	80.41	87.68	40.41	61.80
SD	7.34	5.11	5.37	7.20	13.40	4.86	3.17	2.60	11.15
CV	15.50	10.78	6.38	9.48	20.39	5.62	3.52	6.40	15.89

**Table 3:** Summary of statistical metrics for the linear models of the nine independent CpGs, linear model of the mean of all CpG values, and polynomial model of the mean of all CpG methylation values. The metrics include the coefficient of determination (R^2^), mean absolute error (MAE), root mean square error (RMSE), and *p*-value.

Model	R^2^	MAE	RMSE	*p*-Value
CpG1	0.42	13.57	19.07	2.08 × 10^−11^
CpG2	0.47	12.74	17.35	6.20 × 10^−13^
CpG3	0.48	12.72	17.07	3.30 × 10^−13^
CpG4	0.63	9.84	12.61	3.34 × 10^−19^
CpG5	0.68	8.82	11.07	3.12 × 10^−22^
CpG6	0.62	9.37	12.72	5.26 × 10^−19^
CpG7	0.74	7.54	9.63	8.75 × 10^−26^
CpG8	0.69	8.55	10.94	1.55 × 10^−22^
CpG9	0.72	7.71	10.06	1.23 × 10^−24^
All CpG Linear	0.66	9.42	11.94	6.13 × 10^−21^
All CpG Polynomial	0.69	7.31	9.35	2.20 × 10^−16^

**Table 4:** Summary of the main statistical values obtained from all linear multivariate models. The metrics include the different CpG involved in each model (CpGs Involved), coefficient of determination (R^2^), mean absolute error (MAE), root mean square error (RMSE), and *p*-value.

Model	CpGs Involved	R^2^	MAE	RMSE	*p*-Value
9 CpG	1, 2, 3, 4, 5, 6, 7, 8, 9	0.86	4.55	6.00	1.93 × 10^−28^
8 CpG	1, 2, 3, 4, 6, 7, 8, 9	0.86	4.55	6.01	2.48 × 10^−29^
7 CpG	1, 2, 3, 4, 7, 8, 9	0.86	4.85	6.03	1.07 × 10^−29^
6 CpG	1, 2, 3, 7, 8, 9	0.85	4.89	6.24	5.41 × 10^−30^
5 CpG	1, 3, 7, 8, 9	0.85	4.93	6.30	1.05 × 10^−30^
4 CpG	1, 7, 8, 9	0.83	5.39	6.62	4.67 × 10^−30^
3 CpG	1, 8, 9	0.82	5.67	6.97	2.08 × 10^−29^
2 CpG	1, 9	0.76	6.41	7.97	5.75 × 10^−26^
1 CpG	9	0.72	6.81	8.58	1.23 × 10^−24^

**Table 5:** Statistical values obtained for the comparison between the training model and validation model obtained with a linear multivariate model with eight CpGs non-sex-specific model (CpG1, CpG2, CpG3, CpG4, CpG6, CpG7, CpG8, CpG9). The metrics include the coefficient of determination (R^2^), mean absolute error (MAE) and root mean square error (RMSE), and *p*-value.

Cohort	R^2^	MAE	RMSE	*p*-Value
Training	0.86	4.55	6.01	2.48 × 10^−29^
Validation	0.77	5.04	6.26	1.27 × 10^−17^

**Table 6:** Summary of the main statistical values obtained from all linear multivariate models for females. The metrics include the different CpG involved in each model (CpGs Involved), coefficient of determination (R^2^), mean absolute error (MAE), root mean square error (RMSE), and *p*-value.

Model	CpGs Involved	R^2^	MAE	RMSE	*p*-Value
9 CpG	1, 2, 3, 4, 5, 6, 7, 8, 9	0.87	4.57	6.08	1.29 × 10^−12^
8 CpG	1, 2, 3, 4, 6, 7, 8, 9	0.87	4.57	6.08	2.26 × 10^−13^
7 CpG	1, 3, 4, 6, 7, 8, 9	0.87	4.63	6.15	4.60 × 10^−14^
6 CpG	1, 4, 6, 7, 8, 9	0.87	4.75	5.96	1.05 × 10^−14^
5 CpG	1, 4, 7, 8, 9	0.86	4.74	6.21	2.47 × 10^−15^
4 CpG	4, 7, 8, 9	0.85	4.98	6.48	2.41 × 10^−15^
3 CpG	7, 8, 9	0.79	5.74	7.56	1.52 × 10^−13^
2 CpG	7, 9	0.79	5.72	7.51	1.93 × 10^−14^
1 CpG	7	0.78	5.92	7.63	3.84 × 10^−15^

**Table 7:** Summary of the main statistical values obtained from all linear multivariate models for males. The metrics include the different CpG involved in each model (CpGs Involved), coefficient of determination (R^2^), mean absolute error (MAE), root mean square error (RMSE), and *p*-value.

Model	CpGs Involved	R^2^	MAE	RMSE	*p*-Value
9 CpG	1, 2, 3, 4, 5, 6, 7, 8, 9	0.87	4.12	5.72	2.84 × 10^−11^
8 CpG	1, 2, 3, 4, 6, 7, 8, 9	0.87	4.15	5.76	6.48 × 10^−12^
7 CpG	1, 2, 3, 4, 7, 8, 9	0.87	4.18	5.81	1.92 × 10^−12^
6 CpG	1, 2, 3, 7, 8, 9	0.86	4.22	5.82	4.21 × 10^−13^
5 CpG	1, 2, 3, 7, 9	0.86	4.25	5.91	1.44 × 10^−13^
4 CpG	1, 3, 7, 9	0.84	4.40	6.11	9.44 × 10^−14^
3 CpG	3, 7, 9	0.82	4.69	6.50	1.58 × 10^−13^
2 CpG	3, 7	0.74	5.46	7.64	1.01 × 10^−11^
1 CpG	7	0.71	5.66	7.93	4.90 × 10^−12^

**Table 8:** Statistical values obtained for comparison between the training model and validation model for females and males. The metrics include the coefficient of determination (R^2^), mean absolute error (MAE) and root mean square error (RMSE), and *p*-value.

Cohort	R^2^	MAE	RMSE	*p*-Value
Female training	0.87	4.75	5.96	1.05 × 10^−14^
Female validation	0.72	5.38	7.93	5.22 × 10^−11^
Male training	0.86	4.22	5.82	4.21 × 10^−13^
Male validation	0.78	4.37	5.33	4.71 × 10^−6^

**Table 9:** Statistical values obtained for each age subgroup and comparison with the three models generated. The metrics include the mean absolute error (MAE) and root mean square error (RMSE).

	Eight CpG Non-Sex-Specific Model		Six CpG Female Model		Six CpG Male Model	
CpGs Involved	1, 2, 3, 4, 6, 7, 8, 9		1, 4, 6, 7, 8, 9		1, 2, 3, 7, 8, 9	
Range	MAE	RMSE	MAE	RMSE	MAE	RMSE
20–29	3.67	5.17	5.09	6.40	2.03	2.56
30–39	3.92	5.21	5.21	6.43	3.62	4.28
40–49	3.78	4.74	4.32	5.23	4.24	5.53
50–59	3.09	4.16	3.37	4.16	3.88	5.12
60–69	5.82	6.63	5.97	6.65	3.28	3.82
70–79	7.01	9.36	4.67	6.62	9.40	11.49
Mean	4.55	6.01	4.75	5.96	4.22	5.82

**Table 10:** Population of subjects used in the training group. The means of the ages for each age group are shown, with the standard deviation and the sample size for each one. The sex of the different samples used is also indicated.

	Female		Male	
Age Group	Size (n)	Age (Years)	Size (n)	Age (Years)
20–29	6	24.8 ± 2.3	6	25.0 ± 2.0
30–39	6	33.5 ± 3.4	8	32.9 ± 1.7
40–49	7	43.1 ± 2.1	9	45.1 ± 2.4
50–59	8	54.8 ± 2.6	5	53.4 ± 2.4
60–69	8	64.0 ± 3.0	7	63.3 ± 2.1
70–79	8	73.3 ± 2.6	5	73.4 ± 1.5
Total	43	50.9 ± 17.0	40	47.4 ± 16.2

**Table 11:** Characteristics of subjects used in the validation group. The mean ages for each age group are shown, with the standard deviation and the sample size for each one. The sex of the different samples used is also indicated.

	Female		Male	
Age Group	Size (n)	Age (Years)	Size (n)	Age (Years)
20–29	10	24.7 ± 1.6	6	25.7 ± 2.4
30–39	10	33.7 ± 3.2	4	34.8 ± 3.2
40–49	9	46.0 ± 3.1	5	44.1 ± 1.9
50–59	7	54.4 ± 2.6	1	51
Total	36	38.3 ± 11.5	16	35.4 ± 9.2

## Data Availability

The data presented in this study are available on request from the corresponding author due to the industrial secret with number UV-SECRETO-202487R, requested on 10 October 2024, with co-ownership between Epidisease, S.L., the Consortium Center for Biomedical Research Network, and the University of Valencia.

## Supplementary Materials

The supporting information can be downloaded at: https://www.mdpi.com/article/10.3390/ijms26073392/s1.

## Abbreviations

The following abbreviations are used in this manuscript:

CpG	Cytosine followed by Guanine nucleotide
CV	Coefficient of variation
DNA	Deoxyribonucleic acid
ELOVL2	Elongation of very long chain fatty acids protein 2
MAE	Mean absolute error
miRNA	Non-coding RNA molecules
PCR	Polymerase chain reaction
PUFA	Polyunsaturated fatty acid
R	Coefficient of multiple correlation
R^2^	Coefficient of determination
R^2^ adj	Adjusted coefficient of determination
RNA	Ribonucleic acid
RMSE	Root mean square error
SD	Standard deviation
SEM	Standard error of the mean
