# Supplementary material for: A Sex-Specific Minimal CpG-Based Model for Biological Aging Using ELOVL2 Methylation Analysis
Source: Int J Mol Sci. 2025 Apr 4;26(7):3392. doi: 10.3390/ijms26073392 (PMC11989821; doi:10.3390/ijms26073392)
Supplement: Supplementary file 1 [file ijms-26-03392-s001.zip › ijms-3523816-supplementary.pdf]

Supplementary Table S1. Summary of statistical metrics for the multivariate linear models for all possible combinations using from 1 to 9 CpGs. The CpG combinations are ordered from highest adjusted R^2 to lowest. The metrics include the coefficient of multiple correlation (R), coefficient of determination (R^2), adjusted coefficient of determination (Adjusted R^2), standard error, Mean Absolute Error (MAE), Root Mean Square Error (RMSE), and p-value.

Rank	*CpGs Involved*	*R*	*R²*	*Adjusted R²*	*Standard Error*	*MAE*	*RMSE*	*p-value*
1	*1,2,3,4,6,7,8,9*	*0.93114*	*0.86701*	*0.85264*	*6.36503*	*4.55558*	*6.01003*	*2.48361 x10^{-29}*
2	*1,2,3,4,5,6,7,8,9*	*0.93121*	*0.86715*	*0.85077*	*6.40523*	*4.55406*	*6.00699*	*1.92926 x10^{-28}*
3	*1,2,3,4,7,8,9*	*0.92854*	*0.86219*	*0.84932*	*6.43618*	*4.77736*	*6.11815*	*1.07147 x10^{-29}*
4	*1,2,3,4,5,7,8,9*	*0.92885*	*0.86276*	*0.84792*	*6.46608*	*4.75174*	*6.10545*	*7.85415 x10^{-29}*
5	*1,2,3,6,7,8,9*	*0.92682*	*0.85899*	*0.84583*	*6.51034*	*4.81757*	*6.18864*	*2.50754 x10^{-29}*
6	*1,2,3,5,7,8,9*	*0.92647*	*0.85835*	*0.84513*	*6.52506*	*4.81063*	*6.20263*	*2.96484 x10^{-29}*
7	*1,2,3,7,8,9*	*0.92542*	*0.85640*	*0.84506*	*6.52660*	*4.89288*	*6.24533*	*5.41385 x10^{-30}*
8	*1,3,4,7,8,9*	*0.92527*	*0.85612*	*0.84476*	*6.53284*	*4.86460*	*6.25129*	*5.81774 x10^{-30}*
9	*1,2,3,5,6,7,8,9*	*0.92719*	*0.85968*	*0.84451*	*6.53824*	*4.77950*	*6.17359*	*1.76698 x10^{-28}*
10	*1,3,7,8,9*	*0.92403*	*0.85383*	*0.84434*	*6.54175*	*4.93109*	*6.30087*	*1.05251 x10^{-30}*
11	*1,3,4,6,7,8,9*	*0.92582*	*0.85715*	*0.84382*	*6.55267*	*4.79095*	*6.22888*	*4.05583 x10^{-29}*
12	*1,3,6,7,8,9*	*0.92452*	*0.85473*	*0.84327*	*6.56427*	*4.89400*	*6.28137*	*8.35207 x10^{-30}*
13	*1,3,4,5,7,8,9*	*0.92527*	*0.85612*	*0.84269*	*6.57621*	*4.86615*	*6.25125*	*5.29169 x10^{-29}*
14	*1,3,5,7,8,9*	*0.92421*	*0.85417*	*0.84265*	*6.57708*	*4.89188*	*6.29362*	*9.67311 x10^{-30}*
15	*1,3,4,5,6,7,8,9*	*0.92611*	*0.85767*	*0.84228*	*6.58478*	*4.79174*	*6.21753*	*2.96644 x10^{-28}*
16	*1,3,5,6,7,8,9*	*0.92453*	*0.85475*	*0.84120*	*6.60747*	*4.88890*	*6.28097*	*7.52275 x10^{-29}*
17	*1,2,4,7,8,9*	*0.92289*	*0.85172*	*0.84001*	*6.63208*	*5.09948*	*6.34625*	*1.8114 x10^{-29}*
18	*1,2,4,6,7,8,9*	*0.92383*	*0.85346*	*0.83978*	*6.63686*	*5.05082*	*6.30891*	*1.0456 x10^{-28}*
19	*1,2,4,5,6,7,8,9*	*0.92468*	*0.85504*	*0.83937*	*6.64535*	*5.01378*	*6.27473*	*5.78955 x10^{-28}*
20	*1,2,4,5,7,8,9*	*0.92296*	*0.85185*	*0.83803*	*6.67310*	*5.09106*	*6.34336*	*1.56589 x10^{-28}*
21	*1,4,7,8,9*	*0.92018*	*0.84672*	*0.83677*	*6.69889*	*5.20346*	*6.45222*	*6.46742 x10^{-30}*
22	*1,4,5,7,8,9*	*0.92068*	*0.84765*	*0.83562*	*6.72242*	*5.18064*	*6.43270*	*5.01833 x10^{-29}*
23	*1,4,5,6,7,8,9*	*0.92137*	*0.84893*	*0.83483*	*6.73862*	*5.12405*	*6.40564*	*3.23115 x10^{-28}*
24	*1,4,6,7,8,9*	*0.92025*	*0.84686*	*0.83477*	*6.73979*	*5.18955*	*6.44932*	*6.09491 x10^{-29}*
25	*1,7,8,9*	*0.91557*	*0.83828*	*0.82998*	*6.83676*	*5.39141*	*6.62763*	*4.67399 x10^{-30}*
26	*1,2,7,8,9*	*0.91578*	*0.83866*	*0.82818*	*6.87284*	*5.40196*	*6.61977*	*4.5916 x10^{-29}*
27	*1,5,7,8,9*	*0.91557*	*0.83828*	*0.82778*	*6.88100*	*5.39078*	*6.62762*	*5.0273 x10^{-29}*
28	*1,6,7,8,9*	*0.91557*	*0.83828*	*0.82777*	*6.88101*	*5.39151*	*6.62763*	*5.02771 x10^{-29}*
29	*3,4,6,7,8,9*	*0.91583*	*0.83874*	*0.82601*	*6.91613*	*5.01422*	*6.61806*	*4.2591 x10^{-28}*
30	*1,2,5,7,8,9*	*0.91582*	*0.83873*	*0.82600*	*6.91631*	*5.39667*	*6.61824*	*4.26774 x10^{-28}*
31	*1,2,6,7,8,9*	*0.91580*	*0.83869*	*0.82596*	*6.91726*	*5.39747*	*6.61915*	*4.31205 x10^{-28}*
32	*1,2,3,4,7,9*	*0.91574*	*0.83858*	*0.82584*	*6.91959*	*5.15167*	*6.62138*	*4.42265 x10^{-28}*
33	*1,2,3,4,5,6,7,9*	*0.91803*	*0.84278*	*0.82578*	*6.92068*	*5.10557*	*6.53470*	*1.11963 x10^{-26}*
34	*1,5,6,7,8,9*	*0.91557*	*0.83828*	*0.82551*	*6.92612*	*5.39122*	*6.62762*	*4.74776 x10^{-28}*
35	*1,2,3,4,5,7,9*	*0.91650*	*0.83996*	*0.82503*	*6.93567*	*5.17311*	*6.59296*	*2.73547 x10^{-27}*
36	*1,2,3,7,9*	*0.91401*	*0.83542*	*0.82473*	*6.94159*	*5.27612*	*6.68598*	*9.82511 x10^{-29}*
37	*3,4,5,6,7,8,9*	*0.91620*	*0.83942*	*0.82443*	*6.94742*	*4.99316*	*6.60412*	*3.10068 x10^{-27}*
38	*1,2,3,4,6,7,9*	*0.91607*	*0.83918*	*0.82418*	*6.95255*	*5.11714*	*6.60900*	*3.27498 x10^{-27}*
39	*2,3,4,6,7,8,9*	*0.91585*	*0.83877*	*0.82373*	*6.96143*	*5.01381*	*6.61744*	*3.59995 x10^{-27}*
40	*1,2,5,6,7,8,9*	*0.91583*	*0.83874*	*0.82369*	*6.96220*	*5.39555*	*6.61817*	*3.62926 x10^{-27}*
41	*3,4,7,8,9*	*0.91342*	*0.83433*	*0.82358*	*6.96440*	*5.21432*	*6.70795*	*1.26244 x10^{-28}*
42	*3,6,7,8,9*	*0.91318*	*0.83389*	*0.82311*	*6.97366*	*5.20079*	*6.71688*	*1.3975 x10^{-28}*
43	*1,2,3,5,7,9*	*0.91419*	*0.83574*	*0.82278*	*6.98015*	*5.30282*	*6.67932*	*8.51939 x10^{-28}*
44	*1,2,3,6,7,9*	*0.91412*	*0.83561*	*0.82263*	*6.98294*	*5.26328*	*6.68199*	*8.7796 x10^{-28}*
45	*2,3,4,7,8,9*	*0.91409*	*0.83556*	*0.82258*	*6.98403*	*5.19719*	*6.68304*	*8.88405 x10^{-28}*
46	*2,3,7,8,9*	*0.91279*	*0.83318*	*0.82235*	*6.98859*	*5.27539*	*6.73125*	*1.64555 x10^{-28}*

47	2,3,6,7,8,9	0.91390	0.83521	0.82220	6.99141	5.14393	6.69009	9.61779×10^{-28}
48	2,3,4,5,6,7,8,9	0.91623	0.83948	0.82212	6.99299	4.99213	6.60298	2.38892×10^{-26}
49	1,3,7,9	0.91142	0.83069	0.82201	6.99528	5.48831	6.78130	2.76906×10^{-29}
50	1,3,5,7,9	0.91260	0.83283	0.82198	6.99583	5.47646	6.73823	1.78112×10^{-28}
51	1,3,4,5,7,9	0.91376	0.83496	0.82193	6.99685	5.42254	6.69530	1.0198×10^{-27}
52	3,4,5,7,8,9	0.91355	0.83457	0.82151	7.00498	5.19351	6.70308	1.1129×10^{-27}
53	1,2,3,5,6,7,9	0.91453	0.83636	0.82109	7.01332	5.29765	6.66677	6.23929×10^{-27}
54	3,5,6,7,8,9	0.91325	0.83403	0.82092	7.01652	5.19313	6.71413	1.25966×10^{-27}
55	3,7,8,9	0.91080	0.82955	0.82081	7.01879	5.38705	6.80410	3.59273×10^{-29}
56	1,3,4,5,6,7,9	0.91426	0.83588	0.82056	7.02363	5.41448	6.67657	6.95628×10^{-27}
57	3,5,7,8,9	0.91182	0.83142	0.82047	7.02537	5.30051	6.76668	2.45758×10^{-28}
58	2,3,5,7,8,9	0.91299	0.83355	0.82041	7.02655	5.22479	6.72373	1.40255×10^{-27}
59	2,3,4,5,7,8,9	0.91410	0.83559	0.82024	7.02992	5.18871	6.68255	7.4333×10^{-27}
60	1,3,4,7,9	0.91167	0.83114	0.82017	7.03122	5.46337	6.77231	2.61882×10^{-28}
61	1,3,5,6,7,9	0.91272	0.83306	0.81988	7.03692	5.47577	6.73364	1.567×10^{-27}
62	1,3,6,7,9	0.91150	0.83084	0.81985	7.03749	5.48859	6.77835	2.8034×10^{-28}
63	2,3,5,6,7,8,9	0.91390	0.83522	0.81984	7.03781	5.14410	6.69005	8.07682×10^{-27}
64	1,2,3,8,9	0.91116	0.83022	0.81920	7.05032	5.46235	6.79071	3.2223×10^{-28}
65	1,2,3,4,5,6,7,8	0.91464	0.83656	0.81889	7.05618	5.14985	6.66264	4.60131×10^{-26}
66	1,2,3,4,8,9	0.91184	0.83146	0.81816	7.07057	5.40191	6.76584	2.24337×10^{-27}
67	1,3,4,6,7,9	0.91175	0.83129	0.81797	7.07416	5.46356	6.76928	2.33081×10^{-27}
68	1,2,4,5,6,7,8	0.91278	0.83317	0.81760	7.08131	5.25160	6.73139	1.27453×10^{-26}
69	1,3,4,8,9	0.91024	0.82854	0.81741	7.08512	5.40928	6.82422	4.69352×10^{-28}
70	1,3,4,5,6,7,8	0.91267	0.83297	0.81738	7.08561	5.14829	6.73548	1.33313×10^{-26}
71	1,2,3,6,8,9	0.91135	0.83055	0.81718	7.08959	5.47229	6.78405	2.74589×10^{-27}
72	1,4,5,6,7,8	0.91124	0.83036	0.81697	7.09356	5.22651	6.78784	2.86376×10^{-27}
73	1,2,3,5,8,9	0.91118	0.83024	0.81684	7.09613	5.46325	6.79030	2.94291×10^{-27}
74	1,3,4,6,8,9	0.91081	0.82957	0.81611	7.11016	5.43469	6.80373	3.41429×10^{-27}
75	3,7,9	0.90709	0.82281	0.81608	7.11075	5.56953	6.93729	1.3393×10^{-29}
76	1,2,3,4,6,8,9	0.91198	0.83170	0.81600	7.11240	5.41474	6.76095	1.7628×10^{-26}
77	1,2,3,4,5,8,9	0.91190	0.83156	0.81584	7.11548	5.39233	6.76387	1.82013×10^{-26}
78	3,4,7,9	0.90814	0.82471	0.81572	7.11767	5.50684	6.89995	1.0638×10^{-28}
79	1,4,5,7,8	0.90930	0.82682	0.81558	7.12049	5.30815	6.85830	6.86665×10^{-28}
80	1,3,8,9	0.90798	0.82443	0.81543	7.12335	5.53704	6.90546	1.13176×10^{-28}
81	1,3,4,5,7,8	0.91045	0.82892	0.81541	7.12368	5.24795	6.81666	3.93843×10^{-27}
82	1,3,6,8,9	0.90913	0.82651	0.81524	7.12695	5.56085	6.86451	7.3587×10^{-28}
83	3,4,5,6,7,9	0.91033	0.82869	0.81517	7.12837	5.38192	6.82115	4.13828×10^{-27}
84	1,3,4,5,8,9	0.91027	0.82859	0.81505	7.13060	5.42056	6.82329	4.23689×10^{-27}
85	1,2,3,5,6,8,9	0.91136	0.83057	0.81476	7.13628	5.47082	6.78366	2.25927×10^{-26}
86	1,2,8,9	0.90754	0.82363	0.81459	7.13956	5.63543	6.92118	1.35003×10^{-28}
87	1,3,5,8,9	0.90879	0.82589	0.81459	7.13961	5.55761	6.87671	8.42706×10^{-28}
88	3,4,5,7,9	0.90873	0.82579	0.81448	7.14170	5.53318	6.87873	8.61836×10^{-28}
89	1,2,3,4,5,7,8	0.91118	0.83025	0.81440	7.14313	5.22741	6.79016	2.42541×10^{-26}
90	3,6,7,9	0.90743	0.82343	0.81437	7.14369	5.49858	6.92517	1.41187×10^{-28}
91	1,2,4,5,7,8	0.90986	0.82785	0.81426	7.14596	5.28183	6.83799	4.98093×10^{-27}
92	1,6,8,9	0.90728	0.82316	0.81409	7.14915	5.67200	6.93047	1.49816×10^{-28}
93	2,3,7,9	0.90724	0.82308	0.81401	7.15069	5.56941	6.93197	1.52347×10^{-28}
94	1,8,9	0.90598	0.82080	0.81400	7.15096	5.67074	6.97652	2.08835×10^{-29}
95	1,2,3,4,5,6,8,9	0.91219	0.83209	0.81394	7.15211	5.39187	6.75323	1.23061×10^{-25}
96	1,3,4,5,6,8,9	0.91088	0.82970	0.81381	7.15455	5.42040	6.80102	2.7299×10^{-26}
97	3,5,7,9	0.90712	0.82286	0.81378	7.15515	5.57774	6.93629	1.59895×10^{-28}
98	3,4,6,7,9	0.90836	0.82512	0.81376	7.15545	5.45114	6.89197	9.98178×10^{-28}

99	*1,2,3,4,7,8*	*0.90961*	*0.82739*	*0.81376*	*7.15548*	*5.33304*	*6.84710*	*5.50537 x10^{-27}*
100	*1,3,4,7,8*	*0.90822*	*0.82486*	*0.81349*	*7.16068*	*5.37151*	*6.89701*	*1.0555 x10^{-27}*
101	*2,3,4,7,9*	*0.90815*	*0.82474*	*0.81336*	*7.16326*	*5.50112*	*6.89949*	*1.08491 x10^{-27}*
102	*1,2,6,8,9*	*0.90812*	*0.82468*	*0.81330*	*7.16443*	*5.62408*	*6.90062*	*1.0986 x10^{-27}*
103	*1,2,3,4,6,7,8*	*0.91058*	*0.82915*	*0.81320*	*7.16619*	*5.28197*	*6.81208*	*3.07871 x10^{-26}*
104	*1,5,8,9*	*0.90679*	*0.82228*	*0.81316*	*7.16699*	*5.67049*	*6.94777*	*1.81783 x10^{-28}*
105	*1,3,7,8*	*0.90679*	*0.82227*	*0.81315*	*7.16714*	*5.47683*	*6.94791*	*1.82073 x10^{-28}*
106	*1,3,5,6,8,9*	*0.90926*	*0.82675*	*0.81308*	*7.16865*	*5.55961*	*6.85970*	*6.32172 x10^{-27}*
107	*2,3,4,5,6,7,9*	*0.91039*	*0.82881*	*0.81284*	*7.17324*	*5.34718*	*6.81878*	*3.3111 x10^{-26}*
108	*3,5,6,7,9*	*0.90787*	*0.82423*	*0.81282*	*7.17362*	*5.48333*	*6.90947*	*1.21154 x10^{-27}*
109	*1,2,5,8,9*	*0.90771*	*0.82394*	*0.81250*	*7.17959*	*5.63479*	*6.91522*	*1.29101 x10^{-27}*
110	*1,3,5,7,8*	*0.90765*	*0.82382*	*0.81238*	*7.18193*	*5.43364*	*6.91747*	*1.32351 x10^{-27}*
111	*1,2,4,8,9*	*0.90756*	*0.82366*	*0.81221*	*7.18514*	*5.63139*	*6.92056*	*1.3695 x10^{-27}*
112	*2,3,4,5,7,9*	*0.90879*	*0.82590*	*0.81215*	*7.18634*	*5.54790*	*6.87663*	*7.60877 x10^{-27}*
113	*1,4,8,9*	*0.90625*	*0.82129*	*0.81213*	*7.18677*	*5.67392*	*6.96694*	*2.25099 x10^{-28}*
114	*2,3,5,7,9*	*0.90746*	*0.82348*	*0.81202*	*7.18888*	*5.59151*	*6.92417*	*1.42504 x10^{-27}*
115	*2,3,6,7,9*	*0.90744*	*0.82345*	*0.81198*	*7.18959*	*5.50184*	*6.92485*	*1.43584 x10^{-27}*
116	*1,4,7,8*	*0.90615*	*0.82110*	*0.81193*	*7.19064*	*5.38622*	*6.97070*	*2.3472 x10^{-28}*
117	*1,2,4,7,8*	*0.90740*	*0.82338*	*0.81191*	*7.19100*	*5.38677*	*6.92621*	*1.45755 x10^{-27}*
118	*1,4,6,8,9*	*0.90740*	*0.82337*	*0.81190*	*7.19114*	*5.66197*	*6.92634*	*1.45965 x10^{-27}*
119	*1,5,6,8,9*	*0.90739*	*0.82335*	*0.81188*	*7.19151*	*5.66548*	*6.92670*	*1.46543 x10^{-27}*
120	*1,2,4,5,7,9*	*0.90864*	*0.82563*	*0.81186*	*7.19184*	*5.64025*	*6.88189*	*8.05949 x10^{-27}*
121	*1,3,5,6,7,8*	*0.90858*	*0.82551*	*0.81173*	*7.19431*	*5.38662*	*6.88425*	*8.27009 x10^{-27}*
122	*2,3,4,6,7,9*	*0.90855*	*0.82547*	*0.81169*	*7.19520*	*5.39829*	*6.88510*	*8.34704 x10^{-27}*
123	*1,4,5,7,9*	*0.90724*	*0.82308*	*0.81160*	*7.19694*	*5.73633*	*6.93193*	*1.55237 x10^{-27}*
124	*1,3,4,6,7,8*	*0.90842*	*0.82523*	*0.81143*	*7.20004*	*5.35290*	*6.88973*	*8.77992 x10^{-27}*
125	*1,2,3,7,8*	*0.90712*	*0.82287*	*0.81136*	*7.20140*	*5.46662*	*6.93623*	*1.62768 x10^{-27}*
126	*1,2,4,5,6,7,9*	*0.90956*	*0.82730*	*0.81118*	*7.20485*	*5.59441*	*6.84884*	*4.58452 x10^{-26}*
127	*1,3,6,7,8*	*0.90695*	*0.82256*	*0.81104*	*7.20766*	*5.46373*	*6.94226*	*1.73932 x10^{-27}*
128	*1,2,4,6,8,9*	*0.90813*	*0.82471*	*0.81087*	*7.21087*	*5.62759*	*6.90010*	*9.8307 x10^{-27}*
129	*1,2,5,6,8,9*	*0.90812*	*0.82468*	*0.81084*	*7.21131*	*5.62368*	*6.90053*	*9.8763 x10^{-27}*
130	*1,4,5,8,9*	*0.90682*	*0.82232*	*0.81078*	*7.21253*	*5.67105*	*6.94695*	*1.83134 x10^{-27}*
131	*2,3,5,6,7,9*	*0.90800*	*0.82446*	*0.81061*	*7.21584*	*5.49502*	*6.90485*	*1.0353 x10^{-26}*
132	*1,2,4,6,7,8*	*0.90779*	*0.82409*	*0.81020*	*7.22351*	*5.36335*	*6.91220*	*1.12145 x10^{-26}*
133	*1,2,4,5,8,9*	*0.90777*	*0.82405*	*0.81016*	*7.22434*	*5.62646*	*6.91299*	*1.13113 x10^{-26}*
134	*1,2,3,5,7,8*	*0.90772*	*0.82395*	*0.81005*	*7.22646*	*5.43445*	*6.91501*	*1.15629 x10^{-26}*
135	*1,4,5,6,7,9*	*0.90760*	*0.82373*	*0.80981*	*7.23094*	*5.72127*	*6.91930*	*1.21148 x10^{-26}*
136	*1,2,3,5,6,7,8*	*0.90879*	*0.82590*	*0.80965*	*7.23401*	*5.37700*	*6.87655*	*6.18117 x10^{-26}*
137	*1,2,3,6,7,8*	*0.90751*	*0.82358*	*0.80965*	*7.23403*	*5.43998*	*6.92227*	*1.25108 x10^{-26}*
138	*1,4,6,7,8*	*0.90617*	*0.82115*	*0.80954*	*7.23618*	*5.38336*	*6.96973*	*2.35153 x10^{-27}*
139	*1,4,5,6,8,9*	*0.90744*	*0.82345*	*0.80951*	*7.23674*	*5.65899*	*6.92486*	*1.28675 x10^{-26}*
140	*1,2,4,5,6,8,9*	*0.90814*	*0.82472*	*0.80836*	*7.25845*	*5.62816*	*6.89978*	*7.93268 x10^{-26}*
141	*1,7,8*	*0.90252*	*0.81454*	*0.80749*	*7.27489*	*5.59271*	*7.09742*	*8.08526 x10^{-29}*
142	*1,5,7,8*	*0.90365*	*0.81658*	*0.80717*	*7.28102*	*5.55376*	*7.05831*	*6.185 x10^{-28}*
143	*1,5,6,7,8*	*0.90390*	*0.81703*	*0.80515*	*7.31909*	*5.58055*	*7.04958*	*5.61228 x10^{-27}*
144	*1,2,7,8*	*0.90253*	*0.81457*	*0.80506*	*7.32080*	*5.59423*	*7.09687*	*9.43787 x10^{-28}*
145	*1,6,7,8*	*0.90252*	*0.81455*	*0.80504*	*7.32117*	*5.58830*	*7.09723*	*9.47471 x10^{-28}*
146	*1,2,4,7,9*	*0.90381*	*0.81687*	*0.80498*	*7.32229*	*5.72808*	*7.05267*	*5.80285 x10^{-27}*
147	*1,2,5,7,8*	*0.90370*	*0.81668*	*0.80478*	*7.32607*	*5.55204*	*7.05631*	*6.03604 x10^{-27}*
148	*3,4,5,6,7,8*	*0.90486*	*0.81877*	*0.80447*	*7.33188*	*5.52536*	*7.01589*	*3.43401 x10^{-26}*
149	*1,2,4,6,7,9*	*0.90410*	*0.81740*	*0.80298*	*7.35965*	*5.71679*	*7.04247*	*4.56239 x10^{-26}*
150	*1,2,5,6,7,8*	*0.90393*	*0.81708*	*0.80264*	*7.36597*	*5.57938*	*7.04852*	*4.86623 x10^{-26}*

151	*1,2,6,7,8*	*0.90253*	*0.81457*	*0.80253*	*7.36812*	*5.59150*	*7.09681*	*9.34381 x10^{-27}*
152	*2,3,4,5,6,7,8*	*0.90517*	*0.81933*	*0.80247*	*7.36917*	*5.49722*	*7.00503*	*2.43004 x10^{-25}*
153	*1,5,7,9*	*0.90075*	*0.81135*	*0.80167*	*7.38403*	*5.90479*	*7.15817*	*1.83874 x10^{-27}*
154	*1,4,6,7,9*	*0.90187*	*0.81337*	*0.80125*	*7.39187*	*5.88993*	*7.11968*	*1.19449 x10^{-26}*
155	*1,4,7,9*	*0.90048*	*0.81086*	*0.80116*	*7.39360*	*5.87423*	*7.16744*	*2.03291 x10^{-27}*
156	*2,4,5,7,8,9*	*0.90252*	*0.81454*	*0.79990*	*7.41702*	*5.53608*	*7.09736*	*8.17542 x10^{-26}*
157	*2,4,5,6,7,8,9*	*0.90378*	*0.81682*	*0.79972*	*7.42028*	*5.42704*	*7.05361*	*4.05046 x10^{-25}*
158	*2,4,7,8,9*	*0.90098*	*0.81176*	*0.79953*	*7.42377*	*5.57952*	*7.15041*	*1.65944 x10^{-26}*
159	*1,5,6,7,9*	*0.90087*	*0.81156*	*0.79933*	*7.42758*	*5.91414*	*7.15408*	*1.72576 x10^{-26}*
160	*3,4,6,7,8*	*0.90077*	*0.81138*	*0.79913*	*7.43116*	*5.58836*	*7.15753*	*1.79033 x10^{-26}*
161	*2,3,4,5,7,8*	*0.90213*	*0.81383*	*0.79913*	*7.43119*	*5.63896*	*7.11092*	*9.43565 x10^{-26}*
162	*1,2,5,7,9*	*0.90075*	*0.81136*	*0.79911*	*7.43164*	*5.90147*	*7.15799*	*1.79919 x10^{-26}*
163	*3,4,7,8*	*0.89938*	*0.80888*	*0.79908*	*7.43215*	*5.69732*	*7.20481*	*3.04285 x10^{-27}*
164	*2,3,7,8*	*0.89911*	*0.80840*	*0.79858*	*7.44144*	*5.73612*	*7.21382*	*3.35242 x10^{-27}*
165	*3,4,5,7,8*	*0.90045*	*0.81080*	*0.79852*	*7.44255*	*5.70111*	*7.16850*	*2.0123 x10^{-26}*
166	*1,6,7,9*	*0.89902*	*0.80823*	*0.79840*	*7.44475*	*5.96995*	*7.21703*	*3.47003 x10^{-27}*
167	*2,3,4,7,8*	*0.90031*	*0.81056*	*0.79825*	*7.44743*	*5.64761*	*7.17320*	*2.1156 x10^{-26}*
168	*2,3,5,7,8*	*0.90008*	*0.81015*	*0.79782*	*7.45544*	*5.73878*	*7.18091*	*2.29644 x10^{-26}*
169	*1,7,9*	*0.89720*	*0.80496*	*0.79755*	*7.46034*	*5.95185*	*7.27835*	*5.87319 x10^{-28}*
170	*2,3,5,6,7,8*	*0.90128*	*0.81230*	*0.79748*	*7.46169*	*5.67807*	*7.14011*	*1.28354 x10^{-25}*
171	*2,4,6,7,8,9*	*0.90105*	*0.81189*	*0.79704*	*7.46989*	*5.57100*	*7.14796*	*1.39383 x10^{-25}*
172	*2,3,4,6,7,8*	*0.90101*	*0.81181*	*0.79695*	*7.47138*	*5.58426*	*7.14938*	*1.41491 x10^{-25}*
173	*1,2,5,6,7,9*	*0.90087*	*0.81157*	*0.79669*	*7.47624*	*5.91234*	*7.15403*	*1.48564 x10^{-25}*
174	*3,7,8*	*0.89672*	*0.80410*	*0.79666*	*7.47672*	*5.86693*	*7.29434*	*6.9808 x10^{-28}*
175	*3,6,7,8*	*0.89808*	*0.80655*	*0.79663*	*7.47730*	*5.77714*	*7.24859*	*4.86684 x10^{-27}*
176	*2,3,6,7,8*	*0.89946*	*0.80903*	*0.79663*	*7.47734*	*5.70532*	*7.20201*	*2.87267 x10^{-26}*
177	*1,2,6,7,9*	*0.89918*	*0.80852*	*0.79609*	*7.48734*	*5.94165*	*7.21164*	*3.18098 x10^{-26}*
178	*1,2,7,9*	*0.89778*	*0.80601*	*0.79606*	*7.48786*	*5.92413*	*7.25881*	*5.4291 x10^{-27}*
179	*3,5,6,7,8*	*0.89915*	*0.80847*	*0.79603*	*7.48835*	*5.80619*	*7.21261*	*3.21406 x10^{-26}*
180	*4,5,6,7,8,9*	*0.90036*	*0.81065*	*0.79570*	*7.49441*	*5.54359*	*7.17142*	*1.78284 x10^{-25}*
181	*3,5,7,8*	*0.89681*	*0.80427*	*0.79423*	*7.52138*	*5.87511*	*7.29132*	*7.67648 x10^{-27}*
182	*2,4,5,6,7,8*	*0.89912*	*0.80842*	*0.79330*	*7.53837*	*5.57636*	*7.21349*	*2.76584 x10^{-25}*
183	*4,7,8,9*	*0.89610*	*0.80299*	*0.79289*	*7.54575*	*5.86159*	*7.31494*	*9.86391 x10^{-27}*
184	*4,6,7,8,9*	*0.89731*	*0.80517*	*0.79252*	*7.55248*	*5.72773*	*7.27438*	*6.16056 x10^{-26}*
185	*2,7,8,9*	*0.89580*	*0.80246*	*0.79233*	*7.55605*	*5.64798*	*7.32492*	*1.09646 x10^{-26}*
186	*2,4,5,7,8*	*0.89711*	*0.80481*	*0.79214*	*7.55949*	*5.75862*	*7.28114*	*6.61278 x10^{-26}*
187	*2,4,5,7,9*	*0.89683*	*0.80430*	*0.79159*	*7.56941*	*6.02575*	*7.29068*	*7.30802 x10^{-26}*
188	*4,5,7,8,9*	*0.89678*	*0.80421*	*0.79149*	*7.57120*	*5.85065*	*7.29241*	*7.44109 x10^{-26}*
189	*2,5,7,8,9*	*0.89642*	*0.80357*	*0.79082*	*7.58346*	*5.66555*	*7.30422*	*8.41916 x10^{-26}*
190	*2,4,5,6,7,9*	*0.89768*	*0.80582*	*0.79049*	*7.58933*	*5.98022*	*7.26225*	*4.58669 x10^{-25}*
191	*4,5,6,7,8*	*0.89611*	*0.80302*	*0.79023*	*7.59408*	*5.67113*	*7.31445*	*9.36747 x10^{-26}*
192	*2,6,7,8,9*	*0.89596*	*0.80274*	*0.78993*	*7.59958*	*5.66072*	*7.31975*	*9.89987 x10^{-26}*
193	*4,5,6,7,9*	*0.89568*	*0.80225*	*0.78941*	*7.60893*	*5.94989*	*7.32875*	*1.08729 x10^{-25}*
194	*2,5,6,7,8,9*	*0.89642*	*0.80357*	*0.78807*	*7.63314*	*5.66727*	*7.30417*	*7.06522 x10^{-25}*
195	*4,5,7,9*	*0.89324*	*0.79787*	*0.78751*	*7.64319*	*6.04821*	*7.40940*	*2.66688 x10^{-26}*
196	*1,3,4,6,9*	*0.89461*	*0.80033*	*0.78737*	*7.64569*	*5.76531*	*7.36415*	*1.57034 x10^{-25}*
197	*2,4,7,8*	*0.89311*	*0.79765*	*0.78727*	*7.64743*	*5.75506*	*7.41351*	*2.78399 x10^{-26}*
198	*1,3,4,5,6,9*	*0.89529*	*0.80155*	*0.78588*	*7.67238*	*5.78424*	*7.34172*	*1.03813 x10^{-24}*
199	*1,2,3,4,6,9*	*0.89521*	*0.80140*	*0.78572*	*7.67521*	*5.69796*	*7.34443*	*1.06726 x10^{-24}*
200	*1,3,4,5,9*	*0.89366*	*0.79863*	*0.78556*	*7.67823*	*5.79959*	*7.39550*	*2.17108 x10^{-25}*
201	*2,4,6,7,8*	*0.89313*	*0.79768*	*0.78454*	*7.69634*	*5.74937*	*7.41294*	*2.59847 x10^{-25}*
202	*3,4,5,6,7*	*0.89311*	*0.79764*	*0.78450*	*7.69716*	*6.07240*	*7.41373*	*2.61954 x10^{-25}*

203	1,2,3,4,5,9	0.89445	0.80005	0.78426	7.70133	5.70242	7.36942	1.37712×10^{-24}
204	4,5,7,8	0.89147	0.79472	0.78419	7.70258	5.99988	7.46697	4.85875×10^{-26}
205	2,5,7,8	0.89147	0.79472	0.78419	7.70261	5.84240	7.46700	4.86026×10^{-26}
206	1,3,4,5,6,7	0.89439	0.79993	0.78413	7.70367	6.02941	7.37167	1.40896×10^{-24}
207	1,2,3,4,5,6,9	0.89573	0.80234	0.78389	7.70795	5.72930	7.32707	6.72391×10^{-24}
208	1,2,3,5,9	0.89276	0.79702	0.78384	7.70896	5.80400	7.42510	2.94437×10^{-25}
209	1,3,5,9	0.89119	0.79422	0.78367	7.71200	5.93488	7.47610	5.34173×10^{-26}
210	1,3,5,6,9	0.89256	0.79666	0.78346	7.71564	5.91081	7.43153	3.14521×10^{-25}
211	1,3,4,5,7	0.89252	0.79660	0.78339	7.71690	6.07583	7.43274	3.18456×10^{-25}
212	4,7,9	0.88947	0.79116	0.78323	7.71979	6.15841	7.53148	8.67238×10^{-27}
213	2,4,7,9	0.89090	0.79371	0.78313	7.72156	6.16316	7.48537	5.87978×10^{-26}
214	1,4,5,6,7	0.89227	0.79614	0.78291	7.72553	6.06014	7.44106	3.46815×10^{-25}
215	1,2,3,5,6,9	0.89372	0.79873	0.78284	7.72667	5.81517	7.39367	1.76203×10^{-24}
216	1,4,5,7	0.89072	0.79339	0.78279	7.72759	6.08383	7.49121	6.24646×10^{-26}
217	2,7,8	0.88912	0.79053	0.78257	7.73147	5.86523	7.54287	9.76906×10^{-27}
218	1,2,3,4,5,6,7	0.89505	0.80112	0.78256	7.73169	5.95242	7.34964	8.43725×10^{-24}
219	4,7,8	0.88899	0.79031	0.78235	7.73544	6.08003	7.54674	1.01718×10^{-26}
220	1,2,3,4,9	0.89193	0.79555	0.78227	7.73684	5.66052	7.45195	3.87717×10^{-25}
221	3,4,5,7	0.89028	0.79261	0.78197	7.74215	6.16671	7.50533	7.22733×10^{-26}
222	2,3,4,5,6,7	0.89311	0.79764	0.78166	7.74760	6.07286	7.41370	2.15845×10^{-24}
223	2,5,7,9	0.89005	0.79219	0.78153	7.74998	6.24643	7.51292	7.81657×10^{-26}
224	2,5,6,7,8	0.89154	0.79484	0.78152	7.75012	5.85204	7.46474	4.41884×10^{-25}
225	1,3,4,9	0.88994	0.79200	0.78133	7.75343	5.74656	7.51627	8.09093×10^{-26}
226	1,5,6,9	0.88993	0.79198	0.78132	7.75376	6.05166	7.51659	8.11794×10^{-26}
227	2,4,6,7,9	0.89141	0.79462	0.78128	7.75436	6.14461	7.46882	4.60699×10^{-25}
228	1,2,3,6,9	0.89135	0.79450	0.78115	7.75663	5.82886	7.47101	4.71119×10^{-25}
229	4,6,7,8	0.88983	0.79179	0.78112	7.75732	6.00388	7.52003	8.41124×10^{-26}
230	1,5,9	0.88829	0.78906	0.78105	7.75851	6.13320	7.56925	1.28591×10^{-26}
231	1,2,4,5,6,7	0.89265	0.79682	0.78078	7.76323	5.99083	7.42866	2.51087×10^{-24}
232	1,2,3,4,5,7	0.89265	0.79682	0.78077	7.76336	6.04384	7.42878	2.51392×10^{-24}
233	4,6,7,9	0.88948	0.79117	0.78046	7.76898	6.15714	7.53134	9.45009×10^{-26}
234	2,6,7,8	0.88931	0.79087	0.78015	7.77449	5.84406	7.53668	9.98358×10^{-26}
235	1,2,4,5,7	0.89077	0.79347	0.78005	7.77609	6.06837	7.48975	5.70274×10^{-25}
236	2,3,4,5,7	0.89074	0.79342	0.78000	7.77698	6.18458	7.49062	5.75323×10^{-25}
237	1,3,5,7	0.88899	0.79030	0.77954	7.78510	6.23799	7.54697	1.10964×10^{-25}
238	2,3,4,8,9	0.89040	0.79280	0.77935	7.78854	5.71804	7.50174	6.44252×10^{-25}
239	1,3,6,9	0.88874	0.78986	0.77909	7.79319	6.00953	7.55481	1.20266×10^{-25}
240	1,2,5,9	0.88862	0.78964	0.77885	7.79731	6.10423	7.55880	1.25285×10^{-25}
241	2,5,6,7,9	0.89013	0.79233	0.77885	7.79740	6.24874	7.51028	7.02621×10^{-25}
242	1,2,5,6,9	0.89010	0.79228	0.77879	7.79833	6.04034	7.51118	7.09037×10^{-25}
243	1,4,5,6,9	0.89009	0.79227	0.77878	7.79865	6.06245	7.51148	7.11254×10^{-25}
244	2,3,4,6,8,9	0.89150	0.79477	0.77857	7.80226	5.79170	7.46601	3.6575×10^{-24}
245	1,4,5,9	0.88837	0.78920	0.77839	7.80553	6.12751	7.56678	1.3595×10^{-25}
246	1,3,5,6,7	0.88960	0.79139	0.77784	7.81511	6.24963	7.52734	8.35315×10^{-25}
247	3,5,7	0.88641	0.78572	0.77758	7.81972	6.36555	7.62896	2.38749×10^{-26}
248	2,3,5,7	0.88790	0.78837	0.77751	7.82087	6.34097	7.58164	1.58279×10^{-25}
249	2,6,7,9	0.88771	0.78803	0.77716	7.82710	6.27186	7.58769	1.68361×10^{-25}
250	1,6,9	0.88617	0.78529	0.77714	7.82745	6.13289	7.63651	2.58074×10^{-26}
251	1,4,6,9	0.88765	0.78791	0.77704	7.82924	6.13277	7.58975	1.71951×10^{-25}
252	3,5,6,7	0.88763	0.78789	0.77701	7.82972	6.34900	7.59022	1.7277×10^{-25}
253	2,7,9	0.88609	0.78515	0.77699	7.83008	6.29539	7.63907	2.64982×10^{-26}
254	1,2,3,5,7	0.88907	0.79045	0.77684	7.83272	6.25018	7.54430	9.91672×10^{-25}

255	2,3,4,5,8,9	0.89054	0.79305	0.77672	7.83487	5.73383	7.49720	5.0004 $x10^{-24}$
256	4,5,6,7	0.88745	0.78757	0.77668	7.83551	6.12229	7.59583	1.82955 $x10^{-25}$
257	1,2,4,5,6,9	0.89017	0.79240	0.77601	7.84733	6.05261	7.50913	5.63353 $x10^{-24}$
258	1,2,4,5,9	0.88862	0.78965	0.77599	7.84767	6.10430	7.55870	1.14674 $x10^{-24}$
259	1,2,6,9	0.88706	0.78688	0.77595	7.84833	6.09172	7.60826	2.07661 $x10^{-25}$
260	2,4,5,6,7	0.88859	0.78960	0.77594	7.84853	6.18801	7.55952	1.15634 $x10^{-24}$
261	2,3,5,6,7	0.88854	0.78950	0.77583	7.85047	6.32479	7.56139	1.17834 $x10^{-24}$
262	2,4,5,7	0.88699	0.78675	0.77582	7.85061	6.26741	7.61047	2.12387 $x10^{-25}$
263	2,3,4,5,6,8,9	0.89152	0.79481	0.77566	7.85333	5.78541	7.46526	2.66887 $x10^{-23}$
264	1,2,3,5,6,7	0.88962	0.79142	0.77496	7.86569	6.25769	7.52670	6.71246 $x10^{-24}$
265	1,2,3,9	0.88650	0.78588	0.77490	7.86677	5.83736	7.62614	2.49071 $x10^{-25}$
266	1,2,4,6,9	0.88782	0.78822	0.77447	7.87428	6.11355	7.58433	1.48429 $x10^{-24}$
267	4,5,7	0.88428	0.78195	0.77367	7.88810	6.30239	7.69567	4.73842 $x10^{-26}$
268	1,5,7	0.88316	0.77997	0.77162	7.92381	6.32270	7.73051	6.76195 $x10^{-26}$
269	2,3,5,8,9	0.88576	0.78457	0.77058	7.94182	5.87623	7.64939	2.84598 $x10^{-24}$
270	1,2,5,7	0.88397	0.78140	0.77019	7.94850	6.36384	7.70536	5.54664 $x10^{-25}$
271	2,3,6,8,9	0.88546	0.78405	0.77002	7.95144	5.89914	7.65865	3.1209 $x10^{-24}$
272	3,4,8,9	0.88360	0.78074	0.76950	7.96054	5.94079	7.71704	6.23663 $x10^{-25}$
273	2,3,8,9	0.88351	0.78059	0.76934	7.96328	5.87273	7.71970	6.4056 $x10^{-25}$
274	1,5,6,7	0.88321	0.78006	0.76878	7.97286	6.33270	7.72899	7.03076 $x10^{-25}$
275	2,3,5,6,8,9	0.88634	0.78560	0.76867	7.97479	5.87167	7.63109	1.88501 $x10^{-23}$
276	2,3,4,6,9	0.88413	0.78169	0.76752	7.99469	6.13677	7.70030	4.71804 $x10^{-24}$
277	1,2,5,6,7	0.88398	0.78143	0.76723	7.99954	6.36252	7.70497	4.9412 $x10^{-24}$
278	2,5,7	0.88064	0.77552	0.76700	8.00357	6.48803	7.80833	1.48763 $x10^{-25}$
279	3,4,5,8,9	0.88376	0.78103	0.76682	8.00670	5.91012	7.71188	5.29006 $x10^{-24}$
280	3,4,6,8,9	0.88364	0.78082	0.76659	8.01062	5.94477	7.71565	5.49093 $x10^{-24}$
281	1,2,9	0.87972	0.77390	0.76532	8.03241	6.29626	7.83646	1.97454 $x10^{-25}$
282	2,3,4,5,6,9	0.88451	0.78235	0.76517	8.03494	6.15166	7.68865	3.31021 $x10^{-23}$
283	3,8,9	0.87960	0.77369	0.76510	8.03613	5.99791	7.84010	2.04799 $x10^{-25}$
284	1,4,5,8	0.88121	0.77652	0.76506	8.03673	6.13712	7.79090	1.3042 $x10^{-24}$
285	2,3,4,5,9	0.88278	0.77931	0.76498	8.03818	6.06490	7.74219	7.1328 $x10^{-24}$
286	1,4,9	0.87933	0.77323	0.76461	8.04442	6.36604	7.84819	2.22105 $x10^{-25}$
287	1,2,4,9	0.88085	0.77590	0.76441	8.04791	6.30646	7.80174	1.45256 $x10^{-24}$
288	3,4,5,6,8,9	0.88401	0.78148	0.76423	8.05098	5.93067	7.70401	3.8439 $x10^{-23}$
289	2,5,6,7	0.88064	0.77553	0.76402	8.05459	6.48700	7.80822	1.54895 $x10^{-24}$
290	1,3,9	0.87876	0.77221	0.76356	8.06233	6.08779	7.86566	2.64588 $x10^{-25}$
291	2,3,4,9	0.88033	0.77498	0.76344	8.06452	6.05442	7.81784	1.704 $x10^{-24}$
292	1,5,8	0.87866	0.77205	0.76339	8.06526	6.26561	7.86852	2.72281 $x10^{-25}$
293	2,5,8,9	0.88028	0.77490	0.76336	8.06587	6.07002	7.81915	1.72614 $x10^{-24}$
294	1,4,5,6,8	0.88177	0.77752	0.76307	8.07068	6.13211	7.77350	9.70005 $x10^{-24}$
295	3,5,8,9	0.88001	0.77441	0.76284	8.07462	6.01890	7.82763	1.87728 $x10^{-24}$
296	3,6,8,9	0.88000	0.77440	0.76283	8.07478	6.02262	7.82779	1.88026 $x10^{-24}$
297	2,6,8,9	0.87999	0.77437	0.76280	8.07530	6.17652	7.82829	1.88957 $x10^{-24}$
298	2,4,6,8,9	0.88159	0.77720	0.76273	8.07653	6.13747	7.77913	1.02499 $x10^{-23}$
299	1,2,4,5,8	0.88142	0.77689	0.76241	8.08205	6.14905	7.78445	1.07975 $x10^{-23}$
300	2,5,6,8,9	0.88138	0.77682	0.76233	8.08335	6.10018	7.78570	1.09305 $x10^{-23}$
301	1,3,5,8	0.87956	0.77363	0.76202	8.08854	6.28606	7.84112	2.1452 $x10^{-24}$
302	1,3,4,5,8	0.88121	0.77653	0.76202	8.08859	6.12951	7.79075	1.14842 $x10^{-23}$
303	7,8,9	0.87783	0.77058	0.76186	8.09125	6.35835	7.89387	3.50736 $x10^{-25}$
304	3,4,9	0.87782	0.77056	0.76185	8.09149	6.13715	7.89410	3.51543 $x10^{-25}$
305	2,4,5,8,9	0.88084	0.77587	0.76132	8.10050	6.03970	7.80222	1.28458 $x10^{-23}$
306	3,4,6,9	0.87914	0.77289	0.76125	8.10177	6.20943	7.85395	2.4347 $x10^{-24}$

307	7,9	0.87579	0.76701	0.76119	8.10279	6.55170	7.95501	4.93737 x10^{-26}
308	1,4,6,8	0.87907	0.77277	0.76111	8.10402	6.23444	7.85613	2.48755 x10^{-24}
309	1,5,6,8	0.87901	0.77265	0.76099	8.10609	6.24239	7.85813	2.53714 x10^{-24}
310	2,4,5,6,8,9	0.88220	0.77828	0.76078	8.10965	6.07730	7.76014	6.62159 x10^{-23}
311	3,6,7	0.87722	0.76952	0.76077	8.10982	6.50086	7.91199	4.201 x10^{-25}
312	1,2,4,5,6,8	0.88215	0.77818	0.76067	8.11149	6.15323	7.76190	6.73505 x10^{-23}
313	1,2,5,8	0.87867	0.77207	0.76038	8.11647	6.26155	7.86820	2.8014 x10^{-24}
314	1,9	0.87528	0.76612	0.76027	8.11829	6.41608	7.97022	5.75283 x10^{-26}
315	3,7	0.87521	0.76599	0.76014	8.12045	6.62675	7.97234	5.87681 x10^{-26}
316	6,7,8,9	0.87848	0.77173	0.76002	8.12254	6.35619	7.87408	2.96842 x10^{-24}
317	3,5,6,8,9	0.88012	0.77461	0.75998	8.12327	6.02636	7.82415	1.59077 x10^{-23}
318	1,3,4,5,6,8	0.88178	0.77753	0.75996	8.12347	6.12448	7.77337	7.52229 x10^{-23}
319	3,4,5,9	0.87843	0.77163	0.75992	8.12418	6.17135	7.87568	3.01532 x10^{-24}
320	1,3,5,6,8	0.87999	0.77438	0.75973	8.12746	6.29184	7.82819	1.65445 x10^{-23}
321	5,7,8,9	0.87825	0.77132	0.75960	8.12967	6.32694	7.88100	3.17723 x10^{-24}
322	2,4,8,9	0.87815	0.77114	0.75941	8.13290	6.11743	7.88413	3.27631 x10^{-24}
323	1,2,3,4,5,8	0.88142	0.77690	0.75928	8.13497	6.14390	7.78438	8.36323 x10^{-23}
324	2,8,9	0.87634	0.76797	0.75916	8.13713	6.15072	7.93863	5.47346 x10^{-25}
325	1,2,3,5,8	0.87958	0.77366	0.75897	8.14032	6.29391	7.84058	1.86622 x10^{-23}
326	2,3,7	0.87616	0.76765	0.75883	8.14264	6.49232	7.94401	5.77313 x10^{-25}
327	2,3,5,9	0.87780	0.77053	0.75876	8.14381	6.23686	7.89471	3.63486 x10^{-24}
328	1,2,4,6,8	0.87935	0.77326	0.75853	8.14767	6.26367	7.84765	1.99883 x10^{-23}
329	5,7,9	0.87583	0.76708	0.75824	8.15265	6.54407	7.95378	6.35945 x10^{-25}
330	1,3,4,6,8	0.87918	0.77296	0.75821	8.15306	6.19802	7.85285	2.10223 x10^{-23}
331	6,7,9	0.87581	0.76705	0.75820	8.15325	6.55974	7.95436	6.39592 x10^{-25}
332	3,4,5,6,9	0.87915	0.77291	0.75816	8.15395	6.20901	7.85370	2.11972 x10^{-23}
333	1,3,6,7	0.87733	0.76970	0.75789	8.15846	6.50185	7.90891	4.1774 x10^{-24}
334	1,2,5,6,8	0.87901	0.77265	0.75789	8.15854	6.24186	7.85812	2.21254 x10^{-23}
335	3,4,6,7	0.87731	0.76967	0.75786	8.15896	6.48225	7.90939	4.19711 x10^{-24}
336	7,8	0.87394	0.76377	0.75786	8.15899	6.52143	8.01018	8.58286 x10^{-26}
337	2,3,5,6,9	0.87896	0.77258	0.75781	8.15985	6.27008	7.85939	2.23978 x10^{-23}
338	2,3,6,7	0.87727	0.76960	0.75779	8.16020	6.48704	7.91060	4.24706 x10^{-24}
339	1,2,3,4,5,6,8	0.88215	0.77819	0.75748	8.16535	6.14907	7.76187	4.71204 x10^{-22}
340	1,3,7	0.87538	0.76630	0.75742	8.16640	6.58368	7.96719	7.26131 x10^{-25}
341	3,4,7	0.87521	0.76600	0.75711	8.17157	6.62482	7.97224	7.63207 x10^{-25}
342	5,6,7,8,9	0.87856	0.77187	0.75705	8.17262	6.34309	7.87168	2.52289 x10^{-23}
343	1,4,8	0.87498	0.76559	0.75669	8.17865	6.35594	7.97914	8.16991 x10^{-25}
344	1,2,3,5,6,8	0.88005	0.77449	0.75668	8.17882	6.30572	7.82633	1.2508 x10^{-22}
345	2,3,4,7	0.87655	0.76834	0.75646	8.18256	6.42097	7.93227	5.24921 x10^{-24}
346	1,2,3,7	0.87636	0.76800	0.75611	8.18851	6.49058	7.93803	5.5526 x10^{-24}
347	6,7,8	0.87441	0.76460	0.75566	8.19597	6.47694	7.99604	9.65026 x10^{-25}
348	1,2,3,4,6,8	0.87944	0.77342	0.75554	8.19808	6.22513	7.84477	1.49167 x10^{-22}
349	3,5,9	0.87432	0.76444	0.75549	8.19881	6.35110	7.99881	9.91673 x10^{-25}
350	5,6,7,9	0.87595	0.76729	0.75535	8.20114	6.55770	7.95028	6.25662 x10^{-24}
351	1,2,3,6,7	0.87763	0.77023	0.75531	8.20190	6.45748	7.89989	3.31265 x10^{-23}
352	2,3,4,6,7	0.87748	0.76998	0.75504	8.20636	6.43654	7.90419	3.45265 x10^{-23}
353	1,3,4,6,7	0.87737	0.76979	0.75484	8.20978	6.48758	7.90748	3.56386 x10^{-23}
354	5,7,8	0.87394	0.76377	0.75480	8.21041	6.52154	8.01013	1.10844 x10^{-24}
355	1,3,4,7	0.87542	0.76636	0.75438	8.21740	6.57044	7.96604	7.2932 x10^{-24}
356	1,3,4,8	0.87529	0.76614	0.75415	8.22133	6.29723	7.96985	7.56814 x10^{-24}
357	2,3,6,9	0.87529	0.76613	0.75413	8.22157	6.41250	7.97008	7.58518 x10^{-24}
358	1,2,3,4,7	0.87681	0.76880	0.75379	8.22727	6.40607	7.92432	4.19062 x10^{-23}

359	1,2,4,8	0.87498	0.76559	0.75357	8.23090	6.35640	7.97913	8.28123×10^{-24}
360	3,5,6,9	0.87480	0.76527	0.75323	8.23656	6.37114	7.98462	8.73411×10^{-24}
361	3,6,9	0.87302	0.76217	0.75313	8.23824	6.42485	8.03728	1.44667×10^{-24}
362	5,6,7,8	0.87461	0.76494	0.75289	8.24232	6.47074	7.99020	9.2196×10^{-24}
363	1,2,3,4,6,7	0.87789	0.77069	0.75259	8.24738	6.39942	7.89194	2.33663×10^{-22}
364	2,5,6,9	0.87404	0.76395	0.75184	8.25980	6.43090	8.00715	1.08628×10^{-23}
365	2,5,9	0.87218	0.76069	0.75161	8.26371	6.39395	8.06212	1.84437×10^{-24}
366	1,2,3,4,8	0.87529	0.76614	0.75095	8.27453	6.29730	7.96984	6.48065×10^{-23}
367	2,4,5,6,9	0.87501	0.76564	0.75042	8.28345	6.40385	7.97843	7.03463×10^{-23}
368	1,3,6,8	0.87305	0.76221	0.75002	8.29010	6.46759	8.03652	1.44219×10^{-23}
369	1,6,7	0.87119	0.75897	0.74981	8.29344	6.61152	8.09113	2.44681×10^{-24}
370	2,4,5,9	0.87277	0.76173	0.74951	8.29851	6.40161	8.04467	1.55988×10^{-23}
371	1,4,6,7	0.87238	0.76105	0.74879	8.31038	6.52673	8.05617	1.74229×10^{-23}
372	2,4,6,9	0.87229	0.76088	0.74862	8.31323	6.56386	8.05894	1.78919×10^{-23}
373	5,7	0.86871	0.75466	0.74853	8.31478	6.66198	8.16313	3.89744×10^{-25}
374	1,6,8	0.87039	0.75758	0.74837	8.31734	6.34457	8.11445	3.06861×10^{-24}
375	3,9	0.86845	0.75421	0.74806	8.32240	6.34199	8.17061	4.19384×10^{-25}
376	4,6,7	0.86993	0.75678	0.74754	8.33106	6.55462	8.12783	3.49348×10^{-24}
377	1,2,3,6,8	0.87314	0.76238	0.74695	8.34079	6.44057	8.03366	1.18921×10^{-22}
378	1,2,6,7	0.87123	0.75904	0.74668	8.34524	6.61683	8.08997	2.40886×10^{-23}
379	4,7	0.86766	0.75284	0.74666	8.34553	6.73783	8.19332	5.2364×10^{-25}
380	2,6,9	0.86930	0.75568	0.74640	8.34987	6.64838	8.14618	4.172×10^{-24}
381	2,6,7	0.86926	0.75562	0.74634	8.35084	6.62390	8.14713	4.21028×10^{-24}
382	1,2,6,8	0.87101	0.75866	0.74629	8.35170	6.32528	8.09624	2.55769×10^{-23}
383	5,6,7	0.86906	0.75526	0.74597	8.35693	6.67985	8.15307	4.45886×10^{-24}
384	1,2,4,6,7	0.87254	0.76133	0.74583	8.35927	6.49315	8.05146	1.40729×10^{-22}
385	2,4,6,7	0.87056	0.75787	0.74545	8.36545	6.52097	8.10956	2.90468×10^{-23}
386	2,3,9	0.86854	0.75436	0.74503	8.37235	6.33549	8.16811	5.15481×10^{-24}
387	1,7	0.86673	0.75123	0.74501	8.37274	6.86289	8.22004	6.7944×10^{-25}
388	1,4,7	0.86832	0.75398	0.74464	8.37881	6.78339	8.17442	5.47769×10^{-24}
389	2,4,7	0.86767	0.75286	0.74347	8.39790	6.73406	8.19304	6.55142×10^{-24}
390	1,2,4,7	0.86947	0.75597	0.74346	8.39813	6.70287	8.14124	3.92766×10^{-23}
391	7	0.86339	0.74545	0.74231	8.41695	6.89244	8.31492	8.75351×10^{-26}
392	2,7	0.86515	0.74848	0.74220	8.41877	6.88346	8.26523	1.05352×10^{-24}
393	1,2,7	0.86687	0.75147	0.74203	8.42145	6.85060	8.21601	8.16622×10^{-24}
394	6,7	0.86460	0.74754	0.74123	8.43461	6.78919	8.28077	1.22443×10^{-24}
395	1,3,8	0.86539	0.74889	0.73936	8.46497	6.60715	8.25848	1.22512×10^{-23}
396	1,2,3,8	0.86691	0.75154	0.73879	8.47415	6.62735	8.21494	7.88711×10^{-23}
397	1,2,8	0.86497	0.74817	0.73861	8.47715	6.58155	8.27036	1.37174×10^{-23}
398	2,4,5,8	0.86613	0.75018	0.73736	8.49730	6.44766	8.23738	9.74007×10^{-23}
399	2,3,4,5,8	0.86767	0.75286	0.73681	8.50624	6.34307	8.19302	5.29883×10^{-22}
400	2,4,5,6,8	0.86766	0.75284	0.73679	8.50656	6.48565	8.19332	5.31374×10^{-22}
401	1,8	0.86188	0.74284	0.73641	8.51278	6.52208	8.35752	2.56124×10^{-24}
402	2,3,4,5,6,8	0.86908	0.75530	0.73598	8.51960	6.34656	8.15243	2.65159×10^{-21}
403	2,4,9	0.86298	0.74474	0.73505	8.53468	6.72220	8.32649	2.33548×10^{-23}
404	2,3,4,6,8	0.86635	0.75056	0.73436	8.54573	6.35280	8.23106	7.53599×10^{-22}
405	8,9	0.86051	0.74048	0.73399	8.55175	6.59465	8.39578	3.69089×10^{-24}
406	2,4,6,8	0.86411	0.74668	0.73369	8.55658	6.54675	8.29485	1.66775×10^{-22}
407	2,5,8	0.86188	0.74283	0.73307	8.56653	6.59886	8.35756	3.1308×10^{-23}
408	4,8,9	0.86103	0.74137	0.73155	8.59085	6.57402	8.38128	3.91267×10^{-23}
409	6,8,9	0.86101	0.74133	0.73151	8.59150	6.64502	8.38192	3.93605×10^{-23}
410	5,8,9	0.86081	0.74100	0.73116	8.59708	6.62304	8.38736	4.14225×10^{-23}

411	2,5,6,8	0.86271	0.74427	0.73115	8.59721	6.56354	8.33423	2.40562×10^{-22}
412	2,9	0.85884	0.73761	0.73105	8.59881	6.73114	8.44198	5.72559×10^{-24}
413	4,5,8,9	0.86193	0.74292	0.72974	8.61982	6.58015	8.35616	2.94747×10^{-22}
414	2,3,5,8	0.86190	0.74286	0.72968	8.62074	6.59950	8.35704	2.97181×10^{-22}
415	4,6,8,9	0.86174	0.74259	0.72939	8.62536	6.62093	8.36153	3.09772×10^{-22}
416	5,9	0.85735	0.73505	0.72843	8.64060	6.81821	8.48301	8.43837×10^{-24}
417	5,6,8,9	0.86104	0.74139	0.72813	8.64535	6.64960	8.38090	3.70472×10^{-22}
418	6,9	0.85699	0.73443	0.72780	8.65071	6.87654	8.49293	9.26534×10^{-24}
419	2,3,5,6,8	0.86277	0.74437	0.72777	8.65104	6.56726	8.33249	1.91209×10^{-21}
420	2,3,4,8	0.86038	0.74026	0.72694	8.66433	6.45296	8.39930	4.38952×10^{-22}
421	4,5,6,8,9	0.86208	0.74318	0.72651	8.67118	6.60092	8.35188	2.28165×10^{-21}
422	4,5,9	0.85809	0.73632	0.72630	8.67438	6.80676	8.46278	8.37621×10^{-23}
423	5,6,9	0.85791	0.73602	0.72599	8.67930	6.85113	8.46758	8.75788×10^{-23}
424	9	0.85344	0.72835	0.72500	8.69500	6.81309	8.58960	1.23125×10^{-24}
425	4,6,9	0.85706	0.73456	0.72448	8.70331	6.87627	8.49100	1.0883×10^{-22}
426	2,4,8	0.85669	0.73391	0.72381	8.71383	6.64503	8.50127	1.19684×10^{-22}
427	4,5,6,9	0.85857	0.73714	0.72365	8.71625	6.84363	8.44964	6.96676×10^{-22}
428	4,9	0.85356	0.72857	0.72178	8.74573	6.81355	8.58622	2.22025×10^{-23}
429	2,6,8	0.85145	0.72496	0.71452	8.85916	6.76926	8.64305	4.39498×10^{-22}
430	2,3,6,8	0.85194	0.72580	0.71174	8.90218	6.82894	8.62988	3.56065×10^{-21}
431	3,4,5,8	0.85162	0.72525	0.71117	8.91105	6.65703	8.63847	3.84528×10^{-21}
432	1,2,4,5,6	0.85351	0.72848	0.71085	8.91592	6.97506	8.58762	1.89006×10^{-20}
433	1,4,5,6	0.85052	0.72339	0.70920	8.94124	6.86413	8.66774	4.99375×10^{-21}
434	1,2,4,5	0.85051	0.72337	0.70918	8.94159	6.99078	8.66808	5.00878×10^{-21}
435	2,4,5,6	0.85051	0.72336	0.70918	8.94166	7.11516	8.66815	5.0119×10^{-21}
436	1,4,5	0.84837	0.71973	0.70909	8.94306	6.96154	8.72490	9.22196×10^{-22}
437	3,4,8	0.84796	0.71903	0.70836	8.95423	6.77164	8.73580	1.01729×10^{-21}
438	3,4,6,8	0.85004	0.72257	0.70834	8.95455	6.69983	8.68065	5.60215×10^{-21}
439	3,4,5,6,8	0.85182	0.72561	0.70779	8.96300	6.62636	8.63296	2.8189×10^{-20}
440	3,5,8	0.84741	0.71811	0.70740	8.96890	6.81938	8.75011	1.15699×10^{-21}
441	2,4,5	0.84728	0.71788	0.70717	8.97248	7.08634	8.75360	1.19385×10^{-21}
442	1,2,3,4,5,6	0.85352	0.72849	0.70705	8.97423	6.97707	8.58747	1.28568×10^{-19}
443	2,3,4,5,6	0.85092	0.72406	0.70614	8.98821	7.11520	8.65724	3.48866×10^{-20}
444	1,3,4,5,6	0.85055	0.72344	0.70548	8.99837	6.87043	8.66703	3.8011×10^{-20}
445	1,2,3,4,5	0.85052	0.72339	0.70542	8.99917	6.99139	8.66780	3.82694×10^{-20}
446	1,3,4,5	0.84840	0.71978	0.70541	8.99937	6.96262	8.72410	8.23896×10^{-21}
447	1,5	0.84351	0.71150	0.70429	9.01646	7.19531	8.85201	2.54462×10^{-22}
448	2,3,4,5	0.84775	0.71868	0.70426	9.01696	7.09905	8.74115	9.58008×10^{-21}
449	3,5,6,8	0.84749	0.71824	0.70379	9.02410	6.80225	8.74807	1.01839×10^{-20}
450	5,8	0.84320	0.71099	0.70377	9.02446	7.01857	8.85987	2.7317×10^{-22}
451	1,5,6	0.84511	0.71421	0.70336	9.03069	7.11379	8.81040	1.98496×10^{-21}
452	1,3,5	0.84508	0.71416	0.70330	9.03153	7.13523	8.81122	1.99957×10^{-21}
453	1,3,5,6	0.84689	0.71722	0.70272	9.04037	7.04750	8.76384	1.17046×10^{-20}
454	1,2,3,5,6	0.84839	0.71976	0.70157	9.05789	7.08509	8.72435	6.26852×10^{-20}
455	1,2,5	0.84398	0.71231	0.70138	9.06069	7.21835	8.83967	2.57626×10^{-21}
456	1,2,3,5	0.84611	0.71590	0.70133	9.06151	7.16031	8.78433	1.40176×10^{-20}
457	1,2,5,6	0.84585	0.71546	0.70086	9.06857	7.16688	8.79117	1.48867×10^{-20}
458	5,6,8	0.84334	0.71122	0.70025	9.07788	7.00490	8.85643	2.99011×10^{-21}
459	4,5,8	0.84327	0.71110	0.70013	9.07971	7.01236	8.85823	3.038×10^{-21}
460	2,8	0.84060	0.70660	0.69927	9.09272	6.94030	8.92689	4.99177×10^{-22}
461	2,5	0.84035	0.70619	0.69885	9.09906	7.32174	8.93311	5.27792×10^{-22}
462	2,5,6	0.84238	0.70960	0.69857	9.10324	7.30069	8.88118	3.72332×10^{-21}

463	2,3,5,6	0.84357	0.71161	0.69682	9.12962	7.24806	8.85036	2.4994×10^{-20}
464	2,3,5	0.84125	0.70769	0.69659	9.13304	7.28506	8.91025	4.814×10^{-21}
465	4,5,6,8	0.84342	0.71135	0.69655	9.13369	7.00715	8.85431	2.587×10^{-20}
466	2,3,8	0.84086	0.70705	0.69592	9.14317	6.95550	8.92013	5.25199×10^{-21}
467	6,8	0.83801	0.70226	0.69482	9.15970	7.13032	8.99264	8.97875×10^{-22}
468	4,6,8	0.84014	0.70584	0.69467	9.16192	7.14794	8.93843	6.16982×10^{-21}
469	3,6,8	0.83997	0.70556	0.69437	9.16641	6.94405	8.94280	6.41174×10^{-21}
470	4,8	0.83727	0.70102	0.69355	9.17875	7.21998	9.01134	1.06023×10^{-21}
471	8	0.83311	0.69408	0.69030	9.22731	7.22972	9.11546	1.5514×10^{-22}
472	3,8	0.83429	0.69604	0.68844	9.25501	7.14805	9.08621	2.05539×10^{-21}
473	3,4,5	0.83580	0.69856	0.68711	9.27467	7.31241	9.04843	1.61337×10^{-20}
474	3,4,5,6	0.83695	0.70048	0.68512	9.30412	7.27395	9.01952	1.07794×10^{-19}
475	5	0.82993	0.68878	0.68494	9.30681	7.51076	9.19399	3.11961×10^{-22}
476	2,4,6	0.83453	0.69643	0.68491	9.30730	7.31433	9.08026	2.12611×10^{-20}
477	1,2,4,6	0.83590	0.69873	0.68328	9.33122	7.22818	9.04579	1.34919×10^{-19}
478	2,3,4,6	0.83585	0.69865	0.68320	9.33247	7.28059	9.04701	1.36319×10^{-19}
479	4,5	0.83102	0.69060	0.68286	9.33746	7.44447	9.16716	4.17873×10^{-21}
480	3,5	0.83082	0.69025	0.68251	9.34261	7.46608	9.17221	4.36708×10^{-21}
481	5,6	0.83073	0.69012	0.68237	9.34470	7.47659	9.17426	4.44577×10^{-21}
482	1,4,6	0.83240	0.69289	0.68122	9.36155	7.16837	9.13318	3.35659×10^{-20}
483	4,5,6	0.83194	0.69212	0.68043	9.37324	7.43681	9.14459	3.70262×10^{-20}
484	1,2,3,4,6	0.83660	0.69990	0.68041	9.37345	7.20463	9.02829	8.40925×10^{-19}
485	3,5,6	0.83162	0.69160	0.67988	9.38119	7.44656	9.15234	3.95753×10^{-20}
486	1,3,4,6	0.83328	0.69436	0.67868	9.39877	7.10262	9.11128	2.35386×10^{-19}
487	3,4,6	0.82534	0.68119	0.66908	9.53816	7.37395	9.30548	1.45735×10^{-19}
488	4,6	0.81890	0.67059	0.66236	9.63457	7.58847	9.45885	5.12056×10^{-20}
489	1,3,6	0.80383	0.64614	0.63270	10.04879	8.00663	9.80366	8.74598×10^{-18}
490	3,4	0.80090	0.64144	0.63248	10.05184	7.87691	9.86851	1.5219×10^{-18}
491	2,3,4	0.80212	0.64340	0.62985	10.08766	7.85401	9.84158	1.18408×10^{-17}
492	1,3,4	0.80168	0.64269	0.62912	10.09762	7.83278	9.85129	1.27936×10^{-17}
493	1,2,3,6	0.80391	0.64627	0.62813	10.11118	8.02329	9.80189	6.55776×10^{-17}
494	2,3,6	0.80078	0.64126	0.62763	10.11789	8.12444	9.87108	1.49744×10^{-17}
495	1,4	0.79788	0.63661	0.62752	10.11939	7.96625	9.93482	2.60055×10^{-18}
496	2,4	0.79787	0.63659	0.62751	10.11956	7.96695	9.93499	2.60412×10^{-18}
497	3,6	0.79702	0.63525	0.62613	10.13828	8.06841	9.95337	3.01915×10^{-18}
498	4	0.79415	0.63067	0.62611	10.13849	8.00389	10.01559	3.33836×10^{-19}
499	1,2,3,4	0.80214	0.64342	0.62514	10.15172	7.85205	9.84119	8.92194×10^{-17}
500	1,2,4	0.79839	0.63743	0.62366	10.17169	7.96098	9.92356	2.27003×10^{-17}
501	6	0.79154	0.62654	0.62192	10.19513	8.03865	10.07155	5.25911×10^{-19}
502	1,6	0.79175	0.62687	0.61754	10.25404	8.01664	10.06702	7.48733×10^{-18}
503	2,6	0.79155	0.62654	0.61721	10.25853	8.03929	10.07143	7.75445×10^{-18}
504	1,2,6	0.79232	0.62778	0.61364	10.30620	7.98029	10.05479	6.36213×10^{-17}
505	2,3	0.71085	0.50531	0.49294	11.80682	9.34065	11.59148	5.9341×10^{-13}
506	1,2,3	0.71227	0.50733	0.48862	11.85700	9.34984	11.56777	3.70291×10^{-12}
507	1,3	0.69933	0.48906	0.47629	11.99913	9.41299	11.78028	2.16124×10^{-12}
508	3	0.69450	0.48233	0.47594	12.00318	9.68404	11.85768	3.29987×10^{-13}
509	2	0.68870	0.47431	0.46782	12.09573	9.69418	11.94911	6.19661×10^{-13}
510	1,2	0.68874	0.47436	0.46122	12.17057	9.69962	11.94859	6.72353×10^{-12}
511	1	0.65375	0.42739	0.42032	12.62403	10.06366	12.47101	2.07731×10^{-11}

Supplementary Table S2. Summary of the main statistical values obtained from the linear multivariate model with the 8 CpGs Non-Sex-Specific model. In the multivariate linear model, the intercept and each CpG site variable contribute uniquely to the prediction of the dependent variable. The coefficients represent the magnitude and direction of the relationship between each predictor and the outcome. The probability (p-value) indicates the statistical significance of these relationships.

Variables	Coefficients	Probability
Intercept	-70.99	0.00954
CpG 1	-0.98	0.00017
CpG 2	0.71	0.02185
CpG 3	-1.09	0.00756
CpG 4	-0.84	0.03800
CpG 6	-0.43	0.10546
CpG 7	2.28	0.00003
CpG 8	1.08	0.00019
CpG 9	1.18	0.00002

Supplementary Table S3. Summary of the main statistical values obtained from the linear multivariate model with the 6 CpGs female model (CpG1, CpG4, CpG6, CpG7, CpG8, CpG9). In the multivariate linear model, the intercept and each CpG site variable contribute uniquely to the prediction of the dependent variable. The coefficients represent the magnitude and direction of the relationship between each predictor and the outcome. The probability (p-value) indicates the statistical significance of these relationships.

Variables	Coefficients	Probability
Intercept	-179.85	0.00009
CpG 1	-0.47	0.05751
CpG 4	-1.43	0.02841
CpG 6	-0.71	0.28950
CpG 7	3.53	0.00166
CpG 8	0.99	0.00513
CpG 9	0.95	0.00728

Supplementary Table S4. Summary of the main statistical values obtained from the linear multivariate model with the 6 CpGs male model (CpG1, CpG2, CpG3, CpG7, CpG8, CpG9). In the multivariate linear model, the intercept and each CpG site variable contribute uniquely to the prediction of the dependent variable. The coefficients represent the magnitude and direction of the relationship between each predictor and the outcome. The probability (p-value) indicates the statistical significance of these relationships.

Variables	Coefficients	Probability
Intercept	-33.07	0.24963
CpG 1	-1.19	0.00206
CpG 2	0.47	0.18604
CpG 3	-1.24	0.02772
CpG 7	1.17	0.03193
CpG 8	0.72	0.18976
CpG 9	1.52	0.00308

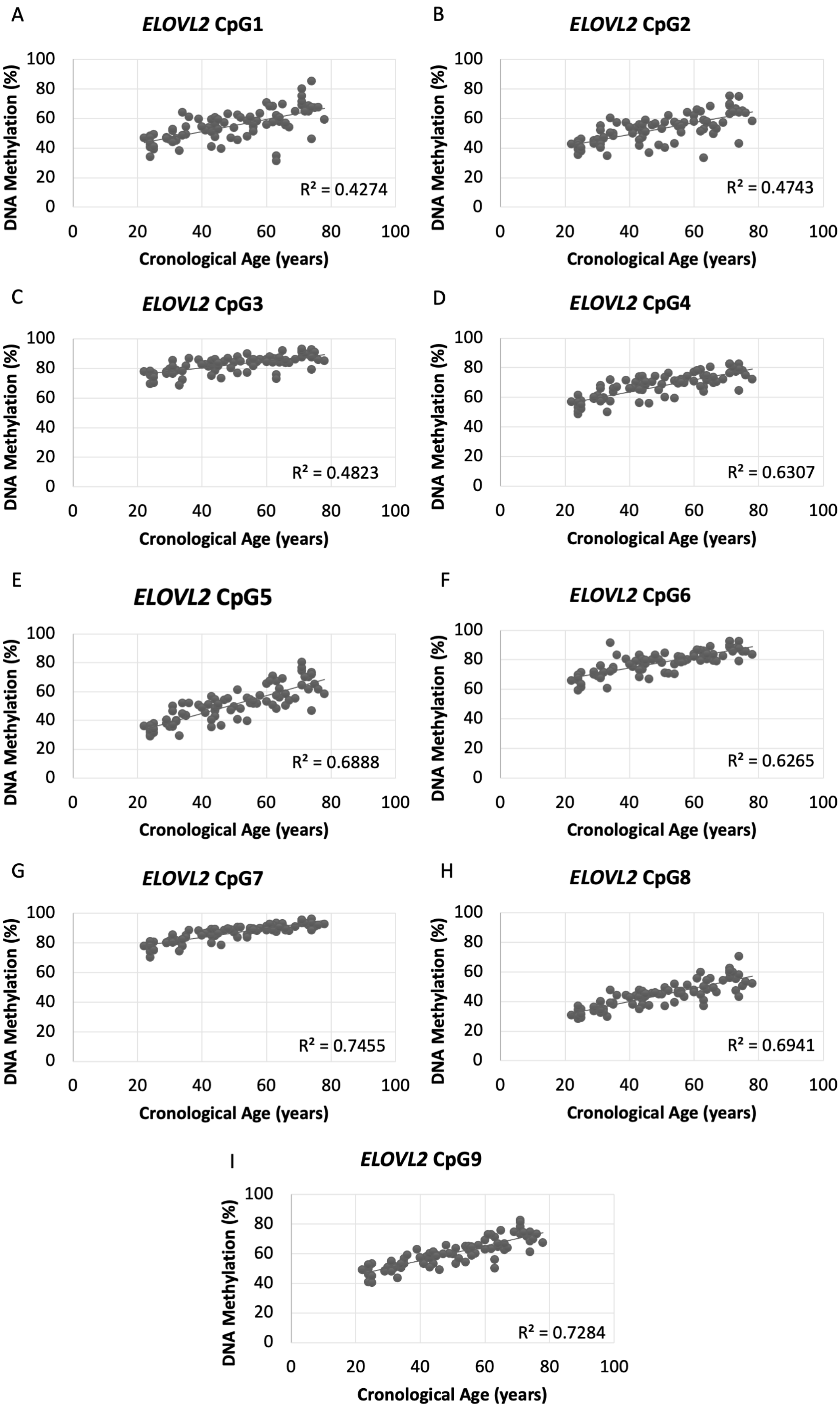

Supplementary Figure S1. Graphical representation of the non-sex-specific linear models of the independent CpGs (A-I). The X-axis represents the chronological values in years, while the Y-axis represents the percentage of DNA methylation. The metric includes the coefficient of determination (R^2).

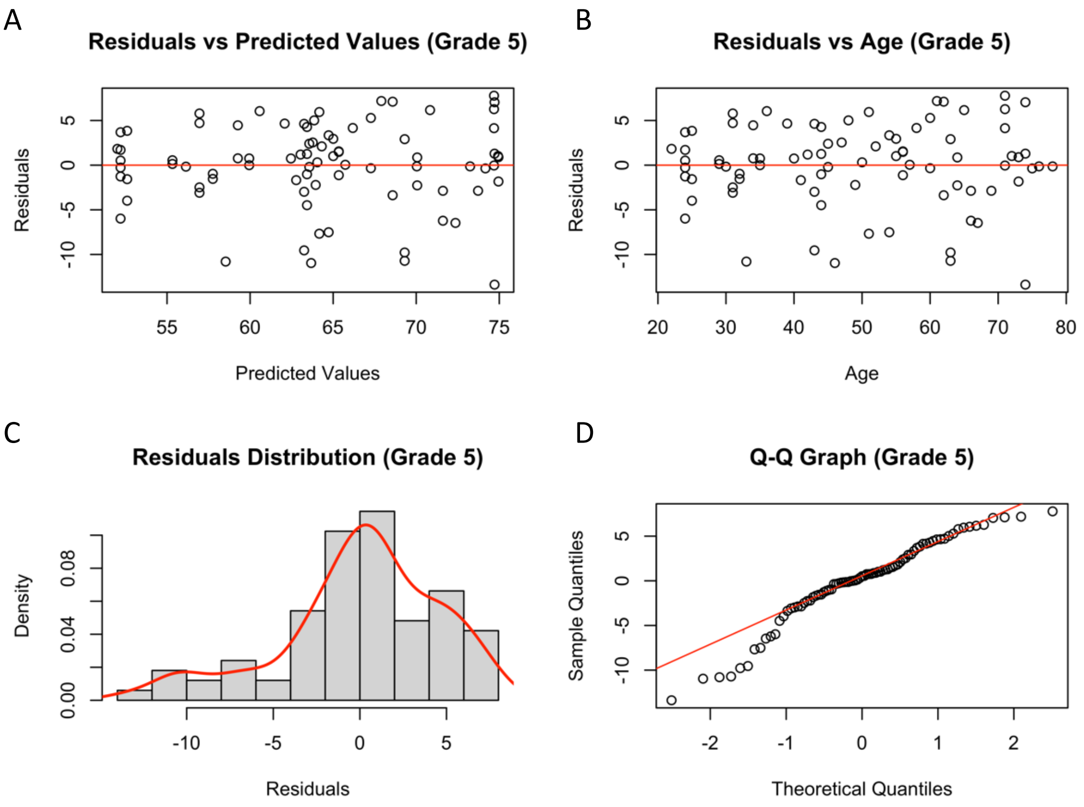

Supplementary Figure S2. Diagnostic plots for the 5th-degree polynomial model of the mean of all CpG methylation values. (A) Residuals vs. predicted values, assessing homoscedasticity. (B) Residuals vs. age, evaluating potential age-related bias. (C) Residuals distribution, visualizing normality assumptions with a density overlay. (D) Q-Q plot, comparing standardized residuals to a normal distribution.
